# Post-amputation reactive oxygen species production is necessary for axolotls limb regeneration

**DOI:** 10.3389/fcell.2022.921520

**Published:** 2022-08-26

**Authors:** Belfran Carbonell-M, Juliana Zapata Cardona, Jean Paul Delgado

**Affiliations:** ^1^ Grupo de Genética, Regeneración y Cáncer, Universidad de Antioquia, Sede de Investigación Universitaria, Medellín, Colombia; ^2^ Departamento de Estudios Básicos Integrados, Facultad de Odontología, Universidad de Antioquia, Medellín, Colombia; ^3^ Grupo de Investigación en Patobiología Quiron, Escuela de MedicinaVeterinaria, Universidad de Antioquia, Medellín, Colombia

**Keywords:** axolotl, reactive oxygen species, hydrogen peroxide, blastema, limb regeneration, *Ambystoma mexicanum*, macrophages, immune cells

## Abstract

**Introduction:** Reactive oxygen species (ROS) represent molecules of great interest in the field of regenerative biology since several animal models require their production to promote and favor tissue, organ, and appendage regeneration. Recently, it has been shown that the production of ROS such as hydrogen peroxide (H_2_O_2_) is required for tail regeneration in *Ambystoma mexicanum*. However, to date, it is unknown whether ROS production is necessary for limb regeneration in this animal model. Methods: forelimbs of juvenile animals were amputated proximally and the dynamics of ROS production was determined using 2′7- dichlorofluorescein diacetate (DCFDA) during the regeneration process. Inhibition of ROS production was performed using the NADPH oxidase inhibitor apocynin. Subsequently, a rescue assay was performed using exogenous hydrogen peroxide (H_2_O_2_). The effect of these treatments on the size and skeletal structures of the regenerated limb was evaluated by staining with alcian blue and alizarin red, as well as the effect on blastema formation, cell proliferation, immune cell recruitment, and expression of genes related to proximal-distal identity. Results: our results show that inhibition of post-amputation limb ROS production in the *A. mexicanum* salamander model results in the regeneration of a miniature limb with a significant reduction in the size of skeletal elements such as the ulna, radius, and overall autopod. Additionally, other effects such as decrease in the number of carpals, defective joint morphology, and failure of integrity between the regenerated structure and the remaining tissue were identified. In addition, this treatment affected blastema formation and induced a reduction in the levels of cell proliferation in this structure, as well as a reduction in the number of CD45^+^ and CD11b + immune system cells. On the other hand, blocking ROS production affected the expression of proximo-distal identity genes such as *Aldha1a1*, *Rarβ*, *Prod1*, *Meis1*, *Hoxa13,* and other genes such as *Agr2* and *Yap1* in early/mid blastema. Of great interest, the failure in blastema formation, skeletal alterations, as well as the expression of the genes evaluated were rescued by the application of exogenous H_2_O_2_, suggesting that ROS/H_2_O_2_ production is necessary from the early stages for proper regeneration and patterning of the limb.

## Introduction

The ability to regenerate structures is widely distributed throughout the animal kingdom including vertebrates and invertebrates (Brockes and Kumar, 2008; Daponte et al., 2021; Elchaninov et al., 2021). Accordingly, understanding the mechanisms underlying this regenerative capacity has become one of the major challenges in developmental and regenerative biology, with the firm purpose of providing new insights and directions for the development of new therapies in the field of regenerative medicine (Brockes and Kumar, 2005; Poss, 2010; Dolan et al., 2018; Iismaa et al., 2018; Arenas Gómez and Echeverri, 2021; Grigoryan, 2021). Within vertebrates, salamanders possess an exceptional ability to regenerate a variety of tissues, organs, and appendages including tail and limbs (Joven et al., 2019). Limb regeneration is a fascinating process and given the complexity of this structure, its regeneration represents one of the main and most conventional models for the study of regenerative response, control of morphogenesis, and final patterning of the regenerated structure (Simon and Tanaka, 2013; Brockes and Gates, 2014; Tanaka, 2016; Dwaraka and Voss, 2019). Among salamanders, Urodeles such as *A. mexicanum* is considered a primary reference model for the study of limb regeneration, given its capacity to regenerate this structure throughout its life (Kragl et al., 2009; Voss et al., 2009, 2015; Haas and Whited, 2017a).

Amputation of a limb in Salamanders including *A. mexicanum* consequently promotes a regenerative response characterized in the first instance by the migration of epithelial cells from the edge of the wound, giving rise to the wound epithelium, which subsequently thickens to form the apical epithelial cap (AEC), which covers the remnant tissue and acts as a signaling center (Tank et al., 1976; Satoh et al., 2012; McCusker et al., 2015; Stocum, 2017; Aztekin, 2021). Simultaneously to the formation of the AEC, other processes such as histolysis and remodeling of the extracellular matrix of the remnant tissue take place, favoring its dedifferentiation, re-entry into the cell cycle, and consequently, the accumulation of progenitor cells (including resident stem cells) between the wound epithelium and the remnant tissue to form the blastema (Tank et al., 1976; McCusker et al., 2015; Voss et al., 2015; Stocum, 2017). Finally, following the growth and patterning of the blastema, a phase of morphogenesis and growth of the regenerating structure takes place to form the lost or amputated limb (McCusker et al., 2015; Voss et al., 2015; Stocum, 2017). During limb regeneration, a wide group of signals has been identified from the immediate amputation to the formation, growth, and patterning of the blastema, as well as signals involved in the morphogenesis of blastema-derived structures (Yokoyama, 2008; Kumar et al., 2010; Makanae and Satoh, 2012; Tanaka, 2016; Haas and Whited, 2017b; Stocum, 2017). Within these signals, we can mention Bioelectrical signals, TGFβ, FGF, SHH, WNT, Retinoic Acid (RA) signaling, and other neurotrophic factors that promote blastema formation and growth, such as n(AG), among others (Borgens et al., 1984; Jenkins et al., 1996; Kawakami et al., 2006; Kumar et al., 2007, 2010; Satoh et al., 2011; Makanae et al., 2014; Nacu et al., 2016; Wischin et al., 2017; Vieira et al., 2019; Maden, 2020; Sader and Roy, 2021). However, other signaling mechanisms are subject to future studies to determine their potential requirement during limb regeneration.

Interestingly, although reactive oxygen species (ROS) have been listed as deleterious molecules for cellular and tissue homeostasis, today they represent molecules of great interest in the field of developmental biology and regeneration, given their capacity to regulate events such as apoptosis, migration, cell proliferation, and differentiation, among other cellular processes (Covarrubias et al., 2008; Hernández-García et al., 2010; Meda et al., 2018; Rampon et al., 2018; Sies and Jones, 2020a). Notably, ROS can regulate the activity of various molecules including transcription factors and kinases (Marinho et al., 2014; Sies, 2017; Rhee et al., 2018)). Previous studies have identified ROS production as a pro-regenerative signal post-amputation of various structures in both vertebrates and invertebrates (Pirotte et al., 2015; Rampon et al., 2018). In vertebrate models, ROS have been extensively involved in the regeneration of appendages such as tail in Gecko (Zhang et al., 2016), tail fin in zebrafish (Yoo et al., 2012; Gauron et al., 2013; Romero et al., 2018; Thauvin et al., 2022), and tail regeneration in *Xenopus* (Love et al., 2013; Ferreira et al., 2016, 2018). Of great interest, in several of these models, it has been evidenced that ROS regulate the activity of signals such as Wnt, Fgf, Shh, and kinases which are described as necessary signals for limb regeneration (Yoo et al., 2012; Love et al., 2013; Romero et al., 2018; Thauvin et al., 2022). In salamanders, studies performed in axolotl embryos show that ROS are necessary for tail regeneration (Al Haj Baddar et al., 2019). In addition, we have recently identified that ROS, particularly H_2_O_2_, promotes tail regeneration in juvenile axolotls by regulating blastema formation and growth through activation of the transcriptional co-activator of the Hippo signaling pathway, Yap1, as well as regulating *Agr2* expression and AKT kinase activation (Carbonell et al., 2021). Of great interest, a recent study in *Xenopus* shows that ROS production induced by melanocortin receptor 4 (*Melanocortin four receptor (Mc4r)*) is necessary to promote limb regeneration (Zhang et al., 2018). However, to date, it is unknown whether ROS production is necessary for axolotl limb regeneration.

Therefore, considering the background of ROS during appendage regeneration in different species, particularly, its implication during tail regeneration in the *A. mexicanum* model, and considering the need to identify whether this signaling mechanism is conserved during regeneration of other structures in this animal model, our aim was to evaluate whether ROS-mediated redox signaling is necessary during axolotl limb regeneration.

Here we show for the first time that post-amputation ROS production is necessary for proper limb regeneration in the *A. mexicanum* model. Thus, ROS production, particularly H_2_O_2_ is required for blastema formation and growth, and blocking NADPH oxidases (NOXs)-dependent ROS production induces regeneration of a miniaturized limb. Additionally, as a first approach to the function of ROS during limb regeneration, it was observed that ROS regulate the expression of several proximal-distal identity genes such as *Meis1, Meis2, Prod1, Hoxa13, Aldh1a1, Rarβ* and other genes such as *Agr2* and *Yap1* during blastema formation. In addition, ROS were necessary to promote the recruitment of CD45 ^+^ leukocytes and CD11b + monocytes/macrophages, as well as their phagocytic activity. These results suggest a potential regulation and interaction between ROS production and these signaling pathways during *A. mexicanum* limb regeneration.

## Materials and methods

### Animal handling and ethical aspects

Leucistic Juvenile axolotls (10 cm snout to tail) were used for this research. Animals were obtained from the *Ambystoma* Genetic Stock Center (AGSC) of the University of Kentucky and bred at the SIU (Sede de Investigación Universitaria) of the University of Antioquia, Colombia. All animals were maintained under the same conditions and fed ad libitum with protein pellets at a temperature between 19 and 21°C in 20% Holftreter’s solution. The animal experimentation procedures were previously approved by the Ethics and Animal Experimentation Committee of the University of Antioquia under the animal experimentation protocol registered in Acta No. 121.

### Limb amputation surgery and regeneration assays

Juvenile axolotls were anesthetized by immersion in 0.1% ethyl-3-aminobenzoate methanesulfonate (Sigma Aldrich, Louis, MO) dissolved in 20% Holtfreter’s solution for all surgical procedures. Bilateral proximal forelimb amputations were performed at the medial humerus level with microsurgical scissors and the protruding bone tissue was regularized with iridectomy scissors to promote proper wound epithelium formation as previously suggested (Kragl and Tanaka, 2009; Arenas Gómez et al., 2017a). Subsequently, the animals were placed in their respective treatments and, once the treatments were completed, they remained in a 20% Holtfreter solution. Each limb was photographed under the same magnification parameters for each of the experimental groups using a stereomicroscope (Olympus SZ×16, Tokyo, Japan) with a digital camera (MotiCAM 5, Kowloon, Hong Kong) pre-amputation, immediate post-amputation, and during the time course of regeneration. The size of the blastema and the regenerated limb was obtained by using Motic Images Plus software (Version 2.0, Kowloon, Hong Kong). Blastema size was determined by subtracting the size of the post-amputation remnant tissue from the distance obtained from the most distal and medial point of the blastema to the most proximal region of the limb at 11, 14, and 21 dpa. The size of the regenerated limbs was determined by subtracting the size of the post-amputation remnant from the distance obtained from the most proximal region of the humerus to the elbow and from the elbow to the most distal point regenerated the second digit at 62 dpa.

### Reactive oxygen species detection

*In vivo* detection of ROS production was performed using 2′7- dichlorofluorescein diacetate (H_2_DCFDA, Sigma). Animals were incubated for 2 h in 50 μM of H_2_DCFDA in a dark environment before image acquisition by confocal microscopy as previously described (Carbonell M et al., 2021). Subsequently, animals were immersed in 40% Holtfreter solution to remove excess H_2_DCFDA and anesthetized in 0.1% ethyl-3-aminobenzoate methanesulfonate (Tricaine) for image acquisition. The animals used for each point were different and were not reused for subsequent analyses. In this way, the potential accumulation of DCFDA residues that could interfere with subsequent detections of ROS production was avoided. Fluorescence generated by oxidation of H_2_DCFDA was acquired on a confocal microscope (Olympus FV1000 MPE, FV10-ASW software) using the mosaic stitching tool given the size of the limb area to be imaged. Samples were excited with a 488 nm laser and the emitted fluorescence was detected using a 520/39 nm filter. Given the thickness of the samples and the difficulty that this can generate to detect the global fluorescence in each sample, a semiquantitative approximation of fluorescence intensity with ImageJ software according to previous studies was performed (Schneider et al., 2012; Jensen, 2013). Thus, a region of interest (ROI) was determined for each acquired image. The area of interest extended from the most distal point of the regenerated tissue to 500 µm proximal to the amputation plane. The intensity of the gray pixels was measured, and the background was subtracted according to previous studies (Schneider et al., 2012; Jensen, 2013; Al Haj Baddar et al., 2019). The gray intensity obtained for each day assessed was compared with pre-amputation levels using a one-way ANOVA and a Tukey’s post hoc analysis between each point assessed was performed to determine differences between these. In addition, fluorescence levels obtained for animals in the control group (0.1% DMSO) and exposed to NOX inhibitors (400 μM apocynin) were compared using Student’s t-test. An n = 6 was used for each point, and a *p*-value < 0.05 was considered statistically significant.

### Chemical inhibition of NOX activity and rescue treatment by exogenous H_2_O_2_ during axolotl’s limb regeneration

For all the tests, the experimental groups remained submerged in each treatment for 1 h before amputation. After the amputation process, animals were incubated in 400 μM apocynin (NOX activity inhibitor, Santa Cruz, SC-203321) for incremental time intervals of 0 dpa - 3 dpa and 0 dpa - 11 dpa. Apocynin inhibits the production of ROS by blocking the formation of the NOX complex (Stefanska and Pawliczak, 2008). Control animals were incubated in 0.1% DMSO for the same time intervals. For the rescue assay, after amputation, animals were incubated from 0 dpa to 11 dpa in 400 μM apocynin combined with 50 μM H_2_O_2_. For the rescue group, a control group exposed to the same concentration of H_2_O_2_ in the absence of the inhibitor was included. A n = 10 animals group was used for each experimental group. Each treatment was changed every 24 h. The size of the regenerated structure was evaluated at 72 dpa and the values obtained were compared by one-way ANOVA and Tukey’s post-hoc analysis to establish the differences between each group evaluated. Similarly, blastema size at 11, 14, and 21 dpa was compared between each group evaluated. A value of *p* < 0.05 was considered statistically significant.

### Histology and immunofluorescence

Regenerated tissues were collected at 11 dpa for histological analysis and stained with Hematoxylin-Eosin (H-E) to evaluate blastema formation and immunofluorescence for cell proliferation assay. Similarly, regenerated limbs were analyzed histologically by H-E staining and Masson’s Trichrome staining at 72 dpa for identification of regenerated tissues including the joint between the stylopod and zeugopod.

After tissue collection, tissues were fixed in 4% paraformaldehyde for 24 h and embedded in paraffin. Subsequently, 5 μm histological sections were stained with H-E or Masson’s Trichrome stain to evaluate the effect of the different experimental conditions. For immunofluorescence, blastemas at 11 dpa from control animals in 0.1% DMSO, exposed to 400 μM apocynin and from the rescue assay group (50 μM H_2_O_2_ + 400 μM apocynin) were collected. Briefly, tissues were fixed in 4% PFA for 24 h, embedded in paraffin, and 5 μm histological sections were made on a microtome. Histological sections were deparaffinized, rehydrated, postfixed in 4% PF4 for 5 min, and washed 3 times for 5 min in 1X TBST (1X Tris-buffered Saline, 0.1% Tween 20). All sections were incubated in blocking solution (10% fetal bovine serum in 1X TBST) for 3 h at room temperature and then incubated with anti-BrdU primary antibody (1:500) diluted in blocking solution at 4°C overnight. The next day, histological sections were washed with 1X TBST and incubated with anti-mouse Alexa fluor 594 secondary antibody (1:200, Abcam #ab 150120). To detect leukocyte recruitment direct immunofluorescence was performed using anti-CD45 FITC Mouse anti-CD45 (1:500, BD Pharmingen), additionally direct immunofluorescence against CD11b was performed using APC mouse anti-CD11b (1:1,000, Invitrogen ref 17-0118-42) to detect monocytes/macrophages. Finally, nuclear counterstaining was performed with Hoechst, and photographs were acquired on an AXIO Vision Zoom V16 stereomicroscope (Carl Zeiss) using ZEN software. An n = 5 was used for each group.

### *In vivo* BrdU labeling assay

5-Bromo-2-deoxyuridine (BrdU) (Sigma, United States) was used as an S-phase proliferative marker during blastema formation. Control animals exposed to 0.1% DMSO (*n* = 5), animals exposed to 400 μM apocynin inhibitor treatment, and animals exposed to rescue treatment (50 μM H2O2 + 400 μM apocynin) were injected with BrdU intraperitoneally (0, 4 mg/g body weight) in two pulses (24 and 48 h before blastema tissue collection) at 9 dpa and 10 dpa according to Arenas *et al* and Carbonell *et al* (Arenas Gómez et al., 2017b; Carbonell et al., 2021). Blastema tissues collected at 11 dpa were processed for immunofluorescence as described in the previous item “histology and immunofluorescence”. A total of five to six histological sections were analyzed for each replicate. The percentage of BrdU-positive cells was determined by dividing the number of BrdU-positive cells by the number of Hoechst-labeled nuclei.

### Skeletal preparations

To visualize skeletal structures, alcian blue and alizarin red staining was performed as previously described (Depew, 2008). Whole limbs were fixed in 4% PFA in 1X PBS for 48 h. Subsequently, the samples were washed in distilled water for 24 h, with water replacement every 8 h. Carefully, skin and muscle tissue were removed with dissecting forceps. The samples were then immersed in 0.3% alcian blue solution for 48 h at 37°C, rehydrated in ethanol series (75%, 40%, and 15%), and washed in distilled water for 2 h. Then, the samples were left in 1% trypsin/sodium borate solution for 24 h and exposed to 0.5% alizarin red/KOH solution for 24 h. Finally, washings were performed with 0.5% KOH, clearing over the next several days by carrying through a 1% KOH/glycerol solution for 24 h at 3:1, 1:1, and 1:3 ratios, and the samples were left in 87% glycerol for photography. Photographs were acquired using stereomicroscopy (Olympus SZX16, Tokyo, Japan) with a digital camera (MotiCAM 5, Kowloon, Hong Kong). Measurements of skeletal structures were performed using Motic Images Plus software (Version 2.0, Kowloon, Hong Kong).

### Phagocytic activity

For the evaluation of phagocytic activity, live neutral red staining was used according to previous studies (Herbomel et al., 2001; Godwin et al., 2013; Franklin et al., 2017). Neutral red stains phagocytic cell populations and has a high affinity for macrophages. This method has been used with great efficiency to label macrophages in zebrafish as well as to identify macrophage phagocytic activity during tail and limb regeneration in *A mexicanum* (Herbomel et al., 2001; Godwin et al., 2013; Franklin et al., 2017)*.* The animals were immersed in neutral red solution 5 μg/ml in Holtfreter’s solution for 6 h. Subsequently, the animals were left for 24 h in Holtfreter’s solution to destain them. Finally, the animals were photographed under a brightfield stereomicroscope with prior anesthesia. Counting of cells per unit area (1mm2) was performed using ImageJ.

### RNA extraction and RT/Q-PCR

Total RNA was isolated from limb blastema at 11 dpa using Trizol reagent (Ambion, 15,596-026) according to the manufacturer’s protocol. Subsequently, cDNA was synthesized using the RevertAid H minus Strand cDNA Synthesis kit (Thermo Scientific #K1632) from 500 ng of total RNA, pretreated with RNase-free DNase I. The cDNA was diluted 1:10 before qPCR assays. The q-PCR reactions were performed using iQ SYBER Green supermix and an iCycler iQTm detection system (Bio-Rad). Gene expression levels were normalized to endogenous 18S reference gene expression as previously reported (Zhu et al., 2012). Three independent experiments (*n* = 3) with respective biological replicates (*n* = 3) and technical triplicates for each experimental point and condition were performed. In addition, negative controls without the first cDNA strand were performed for each gene evaluated. Gene expression levels were calculated by the comparative CT (2^−ΔΔ^CT) method (Schmittgen and Livak, 2008) and between-group comparisons were performed by one-way ANOVA and Tukey’s post-hoc. The sequences of the primers are listed in Table 1 together with the respective reference where they were previously described. All protocols and information necessary for the reproducibility of the experiments will be available to all interested researchers upon request.

**TABLE 1:** List of primers used for Q-PCR.

Gen	Forward 5′-3′	Reverse 5′-3′	References
*Meis1*	CCA​TCT​ACG​GAC​ACC​CCC​T	GGA​AGA​ACA​CAC​GTC​CCC​G	Mercader et al. (2005)
*Meis2*	AGTGGAGGGCACGCTTCT	GCT​TCT​TGT​CTT​TGT​CCG​GGT	Mercader et al. (2005)
*Hoxa13*	TCT​GGA​AGT​CCT​CTC​TGC​CG	TCA​GCT​GGA​CCT​TGG​TGT​ACG	Mercader et al. (2005)
*Prod1*	GGT​GGC​AGT​GAG​CAC​AGG​GT	TGG​CAT​TCC​TGT​ATC​AGA​GT	Shaikh et al. (2011)
*RARA*	ATA​CTT​GGC​AGC​CAG​AAG​GT	GCC​AAC​GTT​GTA​TGC​ATC​TC	Nguyen et al. (2017)
*RARB*	AAA​ACT​CTG​AGG​GGC​TTG​AA	CTG​GTG​TGG​ATT​CTC​CTG​TG	Nguyen et al. (2017)
*RARG*	CTT​CTG​CGT​TTG​ATC​CTT​CA	AGT​GAG​TAT​GGG​GCT​GTT​CC	Nguyen et al. (2017)
*Aldh1a1*	AAG​ACA​TCG​ACA​AGG​CAC​TG	CCA​AAA​GGA​CAC​TGT​GAG​GA	Nguyen et al. (2017)
*Aldh1a2*	GCC​AAG​ACG​GTC​ACA​ATA​AA	CAT​TCC​TGA​GTG​CTG​TTG​CT	Nguyen et al. (2017)
*Yap1*	TAC​CAT​ACC​TTC​CCA​ACA​AAC​C	TAC​ATT​CAT​TGC​TTC​TCC​GTC​T	(Carbonell M et al., 2021)
*Agr2*	GCT​TTC​AAC​AAA​CAC​CTT​CTT​CA	CCTCCACAGAGCCCAGAC	(Carbonell M et al., 2021)
*18S*	AGGCCCTGCCTGCCC	TTA​CGC​TAC​CTT​TGC​ACG​GTC	Zhu et al. (2012)

An illustrative diagram of the Overall experimental design is presented (See Supplementary Figure S1)

## Results

### ROS production during axolotl limb regeneration

**FIGURE 1:**
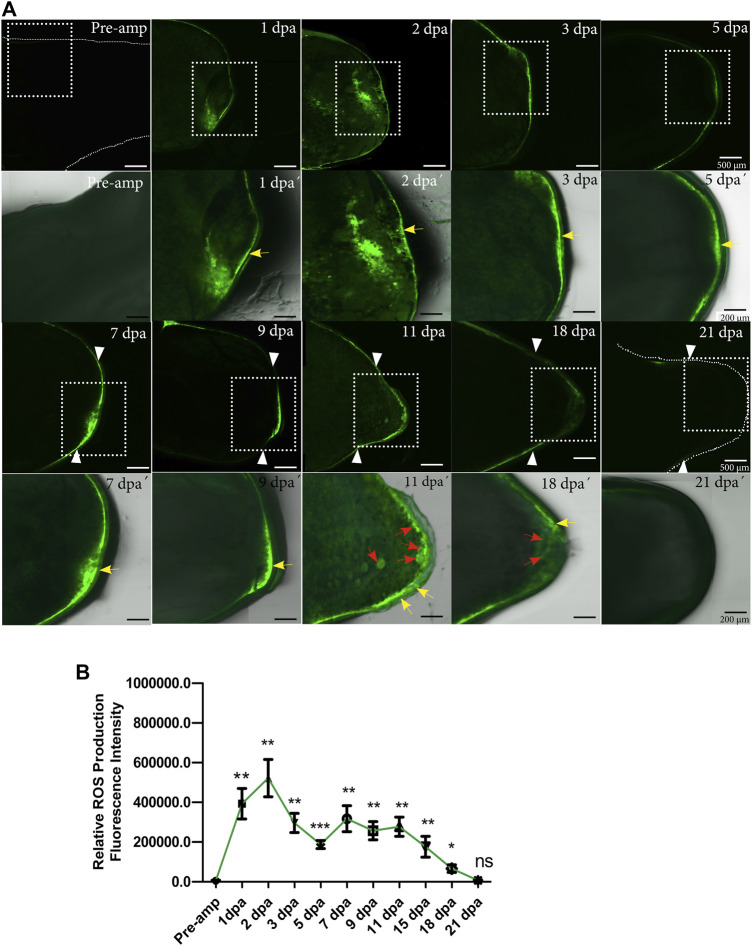
ROS production during limb regeneration in *A. mexicanum*. **(A)** representative images of ROS production acquired by confocal microscopy. The pre-amputation image represents the medial area of the non-amputated humerus (basal level of ROS production). ROS production was detected at the amputation plane at 1, 2, 3, and 5 dpa. At 7 dpa and 9 dpa ROS was detected in the apical epithelial cap (AEC) and at 11 dpa in the AEC and developing blastema region. White arrowheads represent the amputation plane. A white box with a dashed line is shown at higher magnification in lower panel images acquired with differential interference contrast (DIC) in conjunction with fluorescence images for anatomical details. Yellow arrows indicate epithelial localization of ROS and red arrows localization in blastema cells. **(B)**, Semi-quantification of relative fluorescence intensity. Fluorescence levels show two apparent waves of ROS production between 1dpa and 3 dpa for the first wave, and between 7 and 15 dpa, for the second wave of ROS production. One-way ANOVA with a Tukey post-hoc was performed to compare each point with basal levels of pre-amputation ROS production (n = 10 for each point, 5 animals per point). Data are expressed as mean ± SEM (mean standard error). ***p < 0.001, **p < 0.01, *p < 0.05.

As a first step to identify whether ROS are necessary during limb regeneration, we set out to characterize the dynamics of ROS production during the regeneration of this structure using 2′,7′-dichlorofluorescein diacetate (DCFDA). Following limb amputation, at 1 dpa high levels of ROS were detected at the level of the amputation plane and remnant tissue (Figure 1A). ROS production was confined to the *epidermis* covering the wound. These levels increased at 2 dpa maintaining the same localization pattern; however, at 3 dpa and 5 dpa ROS production decreased and ROS production remains localized in the wound epithelium. (Figures 1A,B). At 7 dpa, a new increase in ROS production was detected in the area circumscribed to thickened epithelium covering the wound now called the apical epithelial cap (AEC). These levels were maintained between 9 and 11 dpa when blastema formation takes place. At 9 dpa ROS remained in the AEC and at 11 dpa, in addition to localization in the AEC, signals are detected in presumptive blastema cells (Figure 1A). Finally, ROS levels decreased at 18 dpa reaching basal levels at 21 dpa (Figures 1A,B). Although ROS levels are less detectable at 18 dpa, ROS can be detected in both AEC and presumptive blastemal cells. Fluorescence semiquantification shows that ROS production was detected for at least 18 dpa (Figure 1B). These results indicate that limb amputation significantly induces substantial ROS production during the first 2 weeks post-amputation suggesting a potential role of these molecules during limb regeneration both in the early stages of the regenerative response up to blastema formation and growth.

### NOXs-dependent ROS production is required for proper limb regeneration and exogenous H_2_O_2_ rescues limb regeneration impaired by NOX inhibition

**FIGURE 2:**
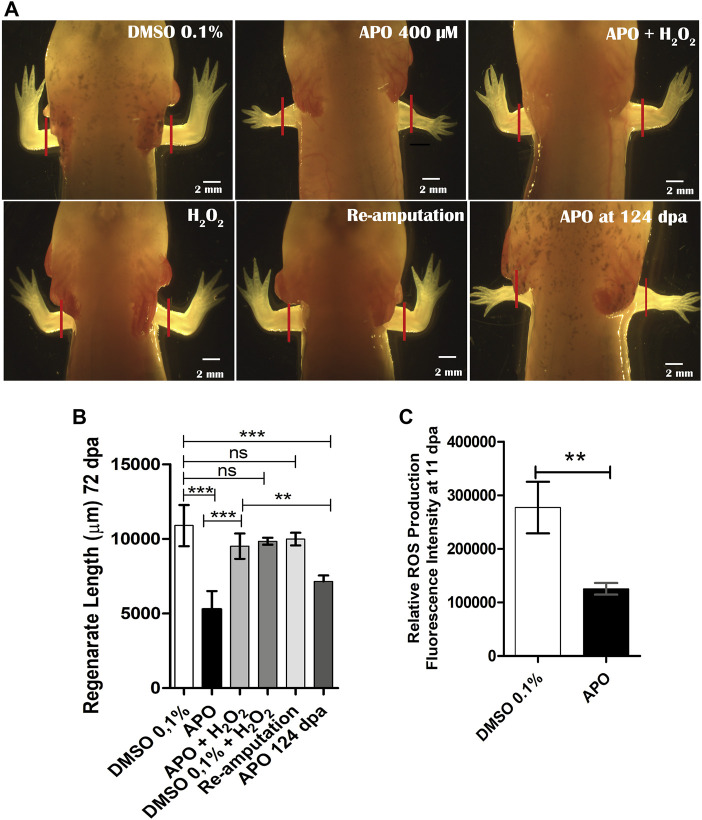
Blocking ROS production by apocynin affects limb regeneration in *A. mexicanum*. **(A)**, Representative images of animals exposed to different treatments compared to the control group in 0.1% DMSO. Defects generated by inhibition of NOX activity and rescue treatments (apocynin 400 µM + H_2_O_2_) are shown. Solid red lines represent the amputation plane. **(B)**, Quantification of regenerated limb size at 72 dpa. Apocynin-exposed group evaluated at 124 dpa was included. One-way ANOVA and post-hoc Tukey were performed to compare the evaluated groups (n = 10 per group). **(C)**, Semi-quantification of ROS levels post-exposure to apocynin inhibitor (*n* = 7). ROS levels decrease significantly under inhibitory treatment. Student’s t-test was performed to compare the two groups. Data are expressed as mean ± SEM. ***p < 0.001, **p < 0.01, *p < 0.05.

To evaluate the requirement of ROS during limb regeneration, we blocked the activity of NOXs using the inhibitor apocynin. Considering the ROS production dynamics characterized above, animals were exposed to 400 µM apocynin and the resulting phenotype was evaluated at 72 dpa. Initially, considering that early blockade of post-amputation tail and caudal fin ROS production in zebrafish larvae and adult animals as well as in *Xenopus* tails and *A mexicanum* affects the regeneration of these appendages (Gauron et al., 2013; Love et al., 2013; Tauzin et al., 2014; Al Haj Baddar et al., 2019; Carbonell M et al., 2021), we hypothesized that given this background and early detection of post-amputation limb ROS production, ROS blockade of 0 dpa-3pa would generate some perturbation in limb regeneration in *A mexicanum.* The animals were incubated in apocynin from 0 to 3 dpa corresponding to the first wave of ROS production; however, the animals regenerated their limbs correctly, without any alteration (*See* Supplementary Figure S2)*.* Subsequently, it was decided to increase the apocynin inhibitor exposure interval from 0 to 11 dpa, covering the second wave of ROS production according to the ROS production dynamics (Figure 1B) and to previous studies showing that extended blockade of ROS production impacts later events during appendage regeneration (Gauron et al., 2013; Love et al., 2013; Carbonell M et al., 2021). The results show that animals exposed to the inhibitor regenerated limbs of reduced size, resembling a miniature structure when compared to control animals exposed to 0.1% DMSO (Figures 2A,B). Additionally, the external morphology of the limbs of apocynin-treated animals showed no apparent intersegmental boundary between the regenerated autopod, zeugopod and stylopod (Figure 2A).

Considering that H_2_O_2_ is one of the main products of NOXs activity and one molecule involved in ROS-mediated redox signaling and that in a previous study we have shown that H_2_O_2_ is required for tail regeneration in *A. mexicanum* (Carbonell M et al., 2021), this prompted us to perform a rescue assay using 50 µM H_2_O_2_ in combination with the inhibitor apocynin from 0 dpa to 11 dpa. The results show that exogenous H_2_O_2_ allows to rescue the phenotype generated by the inhibition of NOXs activity (Figures 2A,B). The regenerated limb in the rescue group presents a larger size compared to animals treated only with the inhibitor apocynin and present an external morphology like that observed in controls exposed to DMSO 0.1%, without apparent syndactyly or supernumerary digits (Figures 2A,B). Animals exposed to exogenous H_2_O_2_+ 0.1% DMSO showed no differences when compared to controls in 0.1% DMSO, indicating that the animals can tolerate this concentration of H_2_O_2_ without affecting the regeneration process (Figures 2A,B). To evaluate whether the defects observed in animals exposed to the inhibitor were permanent, re-amputation of the affected limbs was performed. After re-amputation, the limbs regenerated similarly to controls exposed to 0.1% DMSO (Figures 2A,B). This indicates that the defects generated were the result of transient inhibition of ROS production and that the remaining tissues still retain the ability to induce a regenerative response.

On the other hand, to evaluate whether the regenerated limb with miniature appearance was due to an apparent delay in the speed of the regenerative process post-exposure to the inhibitor, a group of animals exposed to apocynin was evaluated at a longer time corresponding to 124 dpa (approximately twice the time initially evaluated). The results show that this additional time was not sufficient for the miniature limbs to reach a similar size to the controls in DMSO and the rescue group evaluated at 72 dpa (Figures 2A,B). These results suggest that early-stage ROS production potentially influences the determination of the final size of the regenerated structure. Additionally, to ratify that the phenotype observed post apocynin treatment was a product of decreased ROS production, post apocynin treatment, the ROS levels were quantified from 0 dpa to 11 dpa. The results show a significant reduction in ROS levels when compared to the control group (Figure 2C).

**FIGURE 3:**
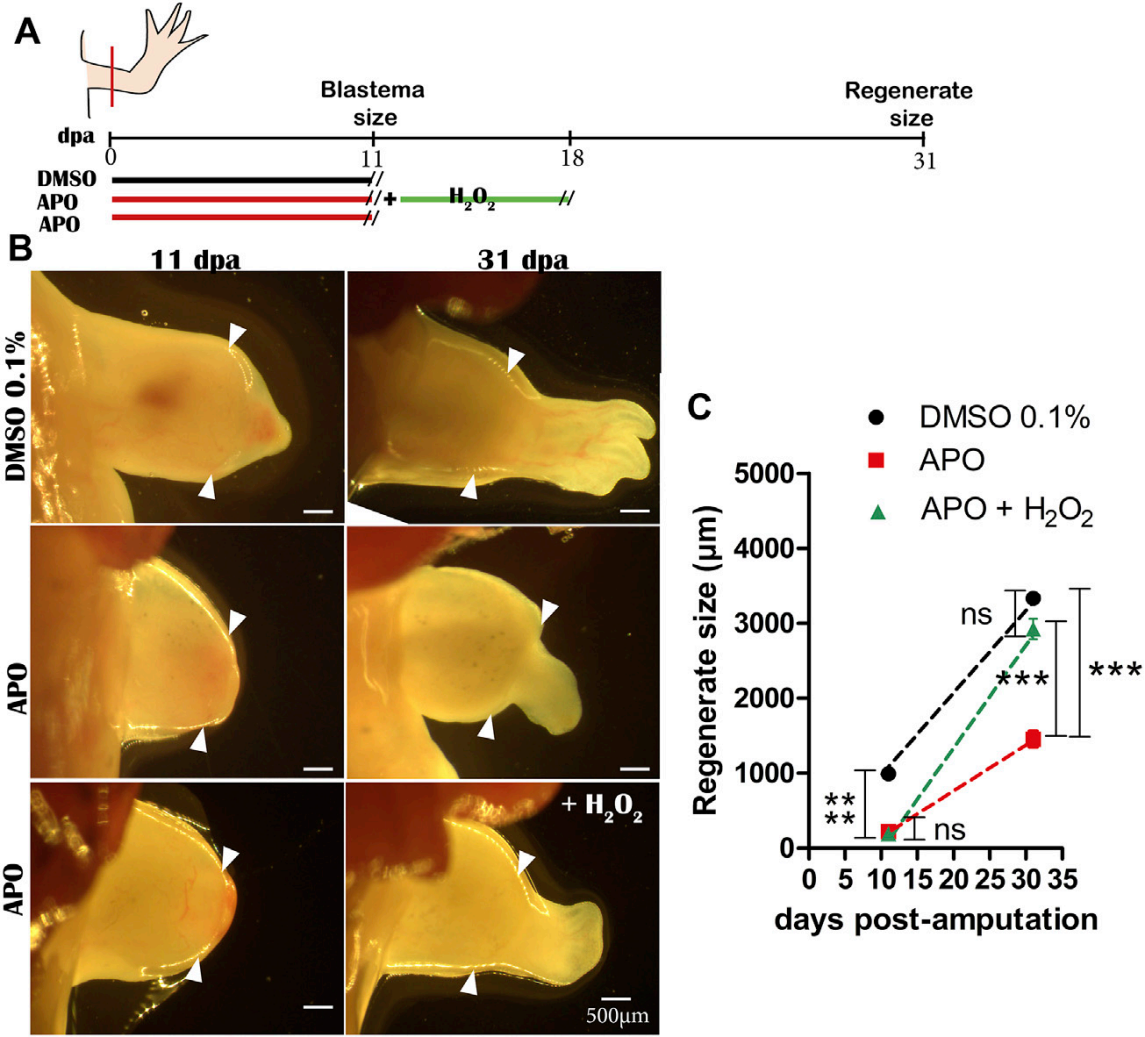
Exogenous H_2_O_2_ induces a rescue effect on the regenerating structure. **(A)** Illustrative scheme of the rescue assay performed. **(B)** Representative images of regenerating tissues at 11 and 31 dpa of controls in 0.1% DMSO, inhibitory treatments with apocynin and without rescue, and inhibitory treatments rescued with exogenous H_2_O_2_. **(C)** Quantification of regenerate size at 31 dpa. White arrowheads represent the amputation plane. One-way ANOVA and post-hoc Tukey were performed to compare the evaluated groups (*n* = 10 per group). Data are expressed as mean ± SEM. ***p < 0.001, **p < 0.01, *p < 0.05.

Finally, to confirm whether the rescue effect observed in the treatments from 0 to 11 dpa was due to H_2_O_2_ as such and not to potential interference between H_2_O_2_ and apocynin, we performed another new rescue assay. Thus, we treated two groups of animals with apocynin from 0 to 11 dpa and withdrew the apocynin treatment. Subsequently, one of these groups previously treated with apocynin was treated with 50 µM exogenous H_2_O_2_ for 7 days until 18 dpa and the other group was not treated at all (Figure 3A). At 31 dpa, we were able to determine that the treatment with 50 µM exogenous H_2_O_2_ effectively promoted a rescue effect on the regenerating structure in contrast to the group not exposed to this treatment, which presented an apparent delay in digit formation and a reduction in the size of the regenerate (Figures 3B,C). This confirms that the rescue effect previously observed when apocynin was administered simultaneously with exogenous H_2_O_2_ was due to the effect of exogenous H_2_O_2_, and not to interference with the inhibitor apocynin (see new Figure 3). This latter result suggests that exogenous H_2_O_2_ can regulate the regenerative response.

### Exogenous H_2_O_2_ rescues skeletal alterations generated by blocking NOX-dependent ROS production

**FIGURE 4:**
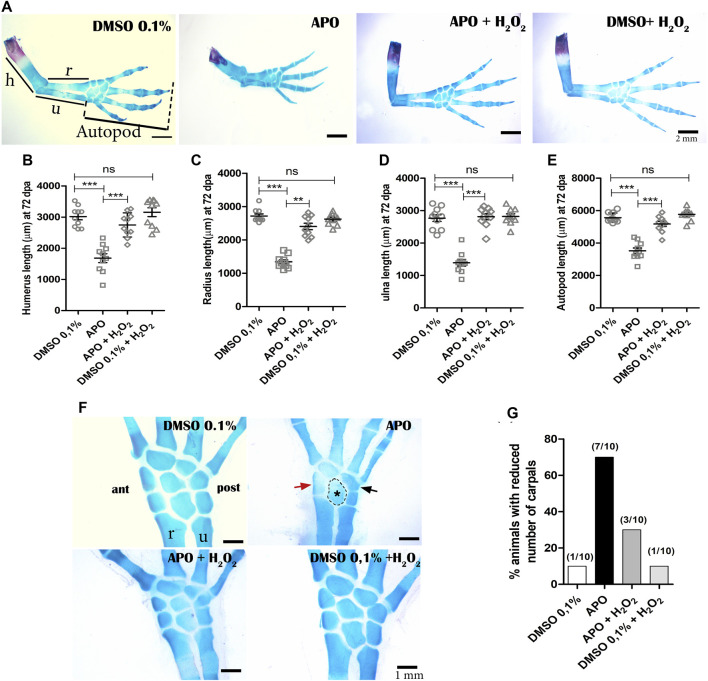
Inhibition of NOXs activity reduces the size of the regenerated skeleton and induces a decrease in the number of carpals in *A. mexicanum* at 72 dpa. **(A)**, Representative images of the treatments evaluated. The skeleton of apocynin (APO)-treated limbs confirms a miniature limb phenotype. Treatments with exogenous H_2_O_2_ partially rescue the phenotype generated by NOXs inhibition. **(B–E)**, Quantification of the skeletal elements of the stylopod (humerus), zeugopod (radius and ulna), and autopod (carpals, metacarpals, and phalanges). One-way and post-hoc Tukey ANOVA was performed to compare the evaluated groups (*n* = 10 per group). **(F)**, inhibition of ROS production by APO generated a decrease in the number of carpals (5 animals had only 5 carpals and 2 animals had six carpals out of 10 animals evaluated). Red arrow, black arrow and dotted circle indicate apparent ectopic fusions in anterior (ant), posterior (post), and intermediate carpals, respectively. **(G)**, Porcetage of animals with reduction in the number of carpals observed in the different experimental groups. h, humerus; r, radius; u, ulna. Data are expressed as mean ± SEM. ***p < 0.001, **p < 0.01, *p < 0.05.

Given our previous observations regarding the post-inhibition effect of ROS production on the size and external morphogenesis of regenerated limbs, we proceeded to perform a skeletal analysis using alcian blue and alizarin red staining. The results showed that animals exposed to apocynin inhibitor from 0 to 11 dpa regenerated the skeletal structures that make up the stylopod (humerus), zeugopod (radius and ulna), and autopod (carpal bones, metacarpals, and phalanges) with a discernible number of four digits (Figure 4A). However, it can be globally identified that the skeleton of the regenerated limb shows a size reduction, recapitulating the external phenotype of a miniature limb described above (Figure 2A). Of great importance, treatment with exogenous H_2_O_2_ was able to rescue the size of the skeleton affected by the inhibition of ROS production, generating a phenotype like that observed in the control group (Figure 4A). A discriminative analysis for each skeletal structure shows that treatment with apocynin generated a proximal-distal reduction in the size of the humerus, radius, ulna, and zeugopodium when compared to the control group in 0.1% DMSO (Figures 4B–E). Similarly, it was identified that the application of exogenous H_2_O_2_ peroxide significantly rescued the size of the affected skeletal structures (Figures 4B–E). These results suggest that redox signaling mediated by ROS/H_2_O_2_ production during the early stages of limb regeneration is necessary to regulate the size of regenerated skeletal structures.

On the other hand, it was identified that inhibition of NOXs activity resulted in a decrease in the number of carpals in 70% of the animals treated with the inhibitor, compared to the controls in 0.1% DMSO, in which a decrease in the number of carpals was identified in 10% of these animals (less than 8 carpals was considered as a reduction in the number of these structures). Of great interest, only 30% of the animals treated with exogenous H_2_O_2_ showed a decrease in the number of carpals, indicating rescue of the phenotype generated by the inhibition of NOXs activity (Figures 4F,G). Additionally, the treatments of the control group exposed to 0.1% DMSO + exogenous H_2_O_2_ did not differ from the control group exposed only to 0.1% DMSO, confirming that the concentration of H_2_O_2_ used does not affect the regeneration process (Figures 4A–G). These results suggest that the inhibition of ROS/H_2_O_2_ production not only affects the proximal-distal size of the regenerate but also disturbs the number of skeletal elements of the autopodium.

**FIGURE 5:**
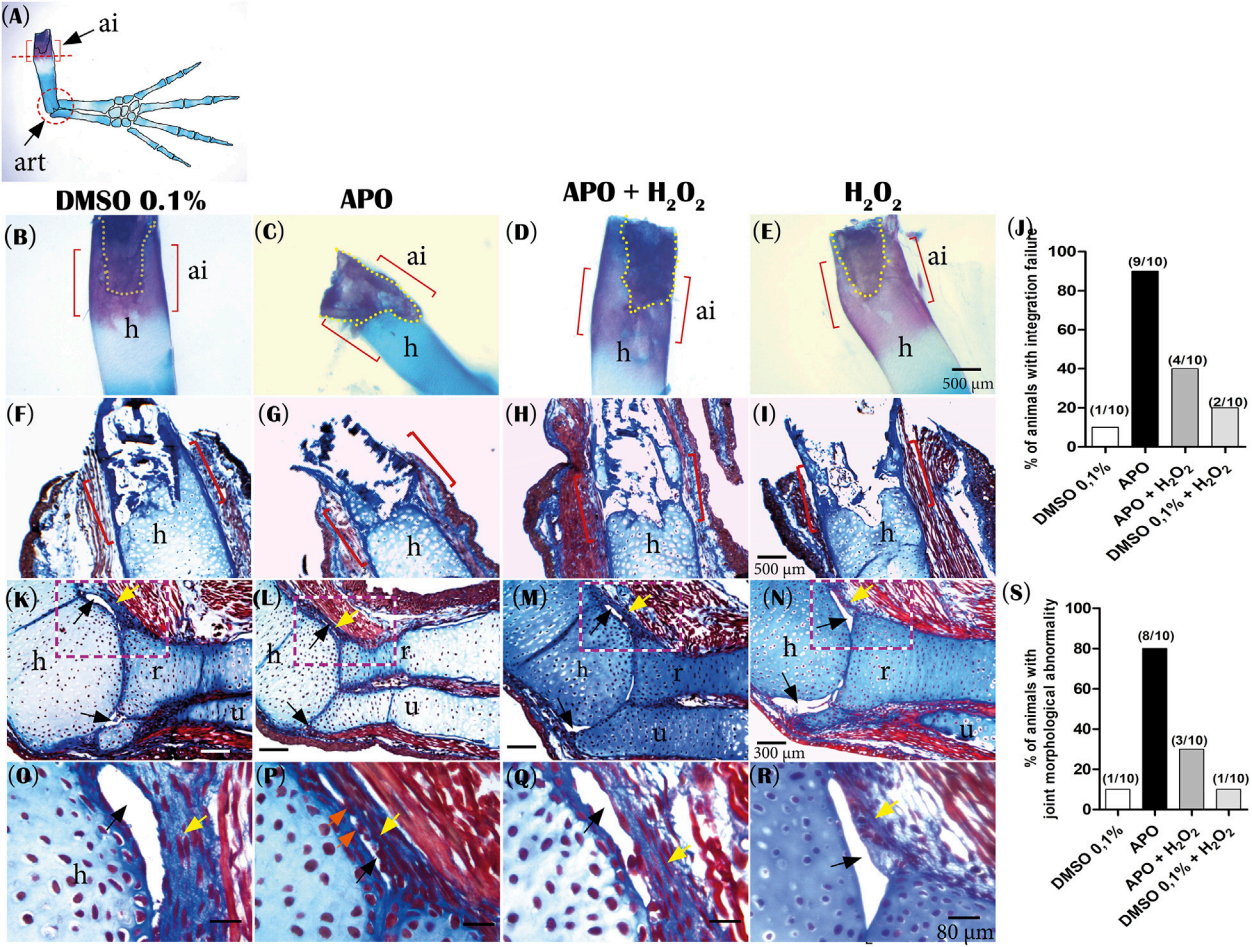
Inhibitory treatment of NOXs-dependent ROS production affects the integration of the regenerated skeleton and joint morphology in *A. mexicanum* at 72 dpa. **(A)**, Illustration of the areas of interest evaluated: ai (integration area) delimited with red brackets. The red circle indicates the joint area studied. **(B–E)**, representative images of the experimental groups evaluated by alcian blue and alizarin red staining: Controls in 0.1% DMSO, apocynin (APO) treatment, rescue assay (APO + H_2_O_2_) and control exposed to 0.1% DMSO +50 µM H_2_O_2_. Yellow dotted lines indicate remnant bone tissue. **(F–I)**, Histological sections with Masson’s trichrome staining at 5 µm. **(F)**, shows continuity between the regenerated humerus and remnant skeletal tissue in the “ai”. **(G)**, the integration area shows discontinuity between the regenerated and remnant humerus. **(H)**, application of exogenous H_2_O_2_ rescues the phenotype affected by ROS inhibition. **(I)**, Control animals exposed to 0.1% DMSO + H_2_O_2_ show integration between regenerated skeletal tissue and remnant skeletal tissue. **(J)**, Percentage of animals that presented integration failure in the different experimental groups. **(K,L)** Representative images of joint morphology. **(K)**, typical synovial joint morphology. **(L)**, APO treatment affects the joint capsule and generates constriction of synovial spaces. **(M)**, treatment with exogenous H_2_O_2_ rescues the articular morphology. **(N),** DMSO + H_2_O_2_ treatments do not affect typical joint morphology. **(O–R)**, magnification of area delimited with red dotted lines in K, L, M, and N. **(O)** A well-defined synovial space can be observed. **(P)** Animals treated with apocynin show a fairly constricted synovial space with apparent continuity between the connective tissue of the articular capsule and the articular surface of the regenerated humerus (orange arrows). **(Q)** Similar to controls, a more defined synovial space is observed. **(R)** An articular morphology as seen in the DMSO controls can be observed. **(S)**, Percentage of animals that presented joint morphological abnormality in the evaluated groups. Yellow arrows indicate connective tissue joint capsule. Black arrows indicate synovial spaces. ai, integration area; h, regenerated humerus; r, radius; u, ulna.

Additionally, we observed that the group of animals treated with the inhibitor apocynin presented failure in the integration between the regenerated skeletal tissue and the remaining skeletal tissue compared to the control group in 0.1% DMSO (Figures 5A–C). For better identification of this defect by using alcian blue and alizarin red staining, we identified a region that we called the “area of integration (ai)”, which corresponds to the area where the regenerated skeletal tissue integrates with the remnant tissue (junction between the most proximal region of the regenerated humerus and the distal region of the remnant humerus) (Figure 5A). Control animals in 0.1% DMSO show an area of integrity characterized by continuity between the remnant humerus and the regenerated humerus (Figure 5B). Histological sections show continuity between the perichondrium of the regenerated humerus, periosteum of the regenerating ossified humerus, and periosteum of the remnant humerus (Figure 5F). In contrast, 90% of the animals treated with apocynin (9/10 animals) present integration failure characterized by the discontinuity between the regenerated humerus and the remnant humerus (Figures 5C,J). This discontinuity can be observed as an angular junction between these two skeletal components; in addition, histological sections show that the perichondrium of the regenerated humerus does not continue with the periosteum of the remaining humerus (Figures 5C,G). Of great importance, rescue treatment with exogenous H_2_O_2_ reduced to 40% (4/10 animals) the integrity failure between the regenerated skeletal tissue and the remnant skeleton generated by inhibition of ROS production (Figures 5D,H). Additionally, only 20% (n = 2/10) of the control animals exposed to 0.1% DMSO + H_2_O_2_ presented integration failure (Figures 5E,I,J). Taken together, these results suggest that ROS production during the first 11 dpa is necessary to promote integration between regenerated skeletal tissue and the remnant skeleton.

Finally, considering that the animals that regenerated miniature limbs did not present a defined boundary between the stylopod and zeugopod, this prompted us to perform an analysis of the joint morphology between these two segments. The results show that control animals exposed to 0.1% DMSO (90%, n = 9/10) presented a typical synovial joint with defined acellular synovial cavities and bounded externally by a connective tissue joint capsule and internally by the articular surfaces of the humerus, radius, and ulna bones (Figures 5K,O,S). In contrast to the controls, the animals treated with apocynin presented an altered articular morphology, characterized by a reduced articular capsule and apparently absent synovial spaces (superior and inferior) (80%, 8/10) (Figures 5L,P,S). On the other hand, animals exposed to rescue treatment with exogenous H_2_O_2_ regenerated a joint with a morphology like controls, and only 30% (*n* = 3/10 joints) presented morphological alterations as those reported in the apocynin-treated group (Figures 5M,Q,S). Additionally, animals exposed to 0.1% DMSO + H_2_O_2_ (90%, *n* = 9/10) presented a typical synovial joint morphology comparable to controls in 0.1% DMSO (Figures 5N,R). These results suggest a potential role of ROS in the early patterning of precursors that will form the articular region between the stylopod and zeugopod.

### NOX-dependent ROS production is required to induce cell cycle re-entry and blastema formation during limb regeneration

**FIGURE 6:**
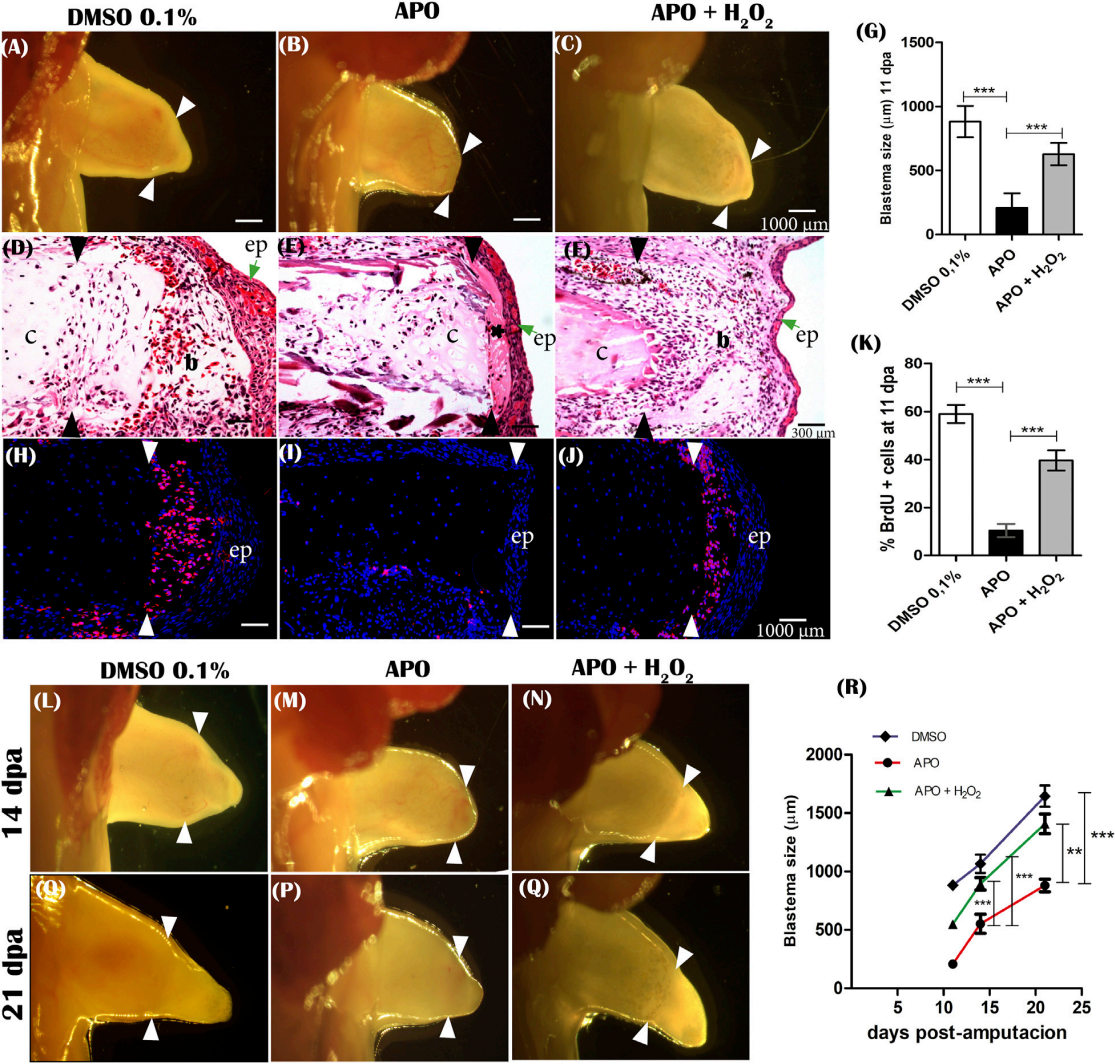
ROS is required for cell cycle re-entry, blastema formation, and blastema growth during limbregeneration in *A. mexicanum*. **(A–C)**, representative images of blastema formation at 11 dpa of controls (DMSO 0.1%), APO and rescue treatment (APO + H_2_O_2_ 50 µM). White arrowheads indicate the amputation plane. **(D–F)**, Hematoxylin-Eosin histologic sections of blastemas at 11 dpa of evaluated groups. **(G)**, Quantification of blastema size. **(H–J)**, Imnunofluorescence anti-BrdU at 11 dpa. Hydrogen peroxide rescues proliferation (re-entry into the cell cycle) of blastema cells affected by inhibition of ROS production. White arrows indicate the amputation plane. **(K)**, Quantification of cell proliferation expressed as percentage of BrdU-positive cells. **(L–Q)**, representative images of blastema growth at 14 dpa and 21 dpa in the different groups evaluated. **(R)**, quantification of blastema size at 14 and 21 dpa. b, blastema; c, cartilage; ep, epithelium.*, indicates area of fibrous tissue accumulation between epithelium and remnant cartilage. Black and white arrowheads indicate the amputation plane. A one-way ANOVA with a Tukey post hoc was performed to compare the groups evaluated (*n* = 10 for blastema sizes and *n* = 5 for immunofluorescence analysis). Data are expressed as mean ± SEM (mean standard error). ***p < 0.001, **p < 0.01, *p < 0.05.

One of the prominent features during limb regeneration is blastema formation. The formation of the blastema and the proliferation of the cells forming this structure are considered fundamental events for the growth of the regenerating structure (McCusker et al., 2015). Therefore, considering that inhibition of ROS production caused a significant reduction in the final size of the regenerated limb, the next step was to evaluate the effect of inhibition of NOXs activity on blastema formation. The results show that at 11 dpa, control animals in 0.1% DMSO formed an early blastema characterized by the accumulation of mesenchymal cells between the apical epithelial layer and the remnant cartilage (Figures 6A,D). In contrast, the apocynin-treated group of animals from 0 to 11 dpa showed an apparent absence of blastema formation (Figure 6B). Histological sections show sparse blastema cells between the epithelium and remnant cartilage in this experimental group (Figure 6E). Of great interest, between the epithelial layer and the remnant cartilage, a predominant deposition of connective tissue was identified overlying the accumulation of blastema mesenchymal cells (Figure 6E). Accordingly, we decided to evaluate whether the application of exogenous H_2_O_2_ could rescue blastema formation. The results show that exogenous H_2_O_2_ from 0 to 11 dpa induced the formation of a blastema similar in size and shape to the control group, favoring the accumulation of mesenchymal cells between the epithelium and the remnant cartilage (Figures 6C,F,G). This shows that exogenous H_2_O_2_ is sufficient to rescue the size of the blastema affected by ROS inhibition. Finally, although at 11 dpa blastema formation was affected by apocynin treatments, we followed up with these animals and identified the formation of a late blastema whose size was smaller compared to the control group in DMSO at 14 and 21 dpa. However, blastema growth in the rescue group animals experienced similar growth to the control group in DMSO (Figures 6L–R). These results show that although there is a delayed blastema formation in apocynin-treated animals, its size was smaller compared to controls, which correlates with the final size and miniaturized appearance of the regenerated limb.

Additionally, considering the deficient blastema formation in apocynin-treated animals, the effect of ROS inhibition on blastema cell proliferation by BrdU incorporation was evaluated. The results show that apocynin-treated animals showed a significant reduction in the percentage of cells incorporating BrdU compared with the control group in 0.1% DMSO (Figures 6H,I). Few BrdU-positive cells were detected at the amputation plane, whereas only a small percentage of positive cells were detected lateral to the amputation plane in apocynin-treated animals (Figure 6I). Of great importance, rescue treatment with exogenous H_2_O_2_ increased BrdU incorporation levels like controls in 0.1% DMSO. Therefore, H_2_O_2_ can rescue the effect generated by inhibition of ROS production (Figures 6J,K). These results suggest that ROS production is required for cell cycle re-entry and subsequent blastema formation and growth during limb regeneration in axolotls.

### Identifying potential target genes regulated by ROS production during axolotl limb regeneration

In order to approach a potential explanation for the phenotypic alterations observed upon inhibition of ROS production such as failure of blastema formation, shortening of regenerated skeletal structures, decrease in the number of carpals, alteration in joint morphogenesis and failure of the integrity of the new regenerated structure to the remnant, we set out to evaluate the effect of ROS on the expression of genes related to blastema formation and growth in axolotls, we set out to evaluate the effect of ROS on the expression of genes related to blastema formation and growth (*Agr2* and *Yap1*), positional identity and tissue integrity such as Retinoic Acid (RA) pathway genes such as *Aldh1a1, Aldh1a2, Rarβ, Rarα, RarG*, and other positional identity genes such as *Meis1, Meis2, Prod1* and *Hoxa13*. Accordingly, the expression of these genes was evaluated by RT-qPCR after blocking NOX-dependent ROS production and after exposure to rescue assay with exogenous H_2_O_2_ from 0 to 11 dpa.

**FIGURE 7:**
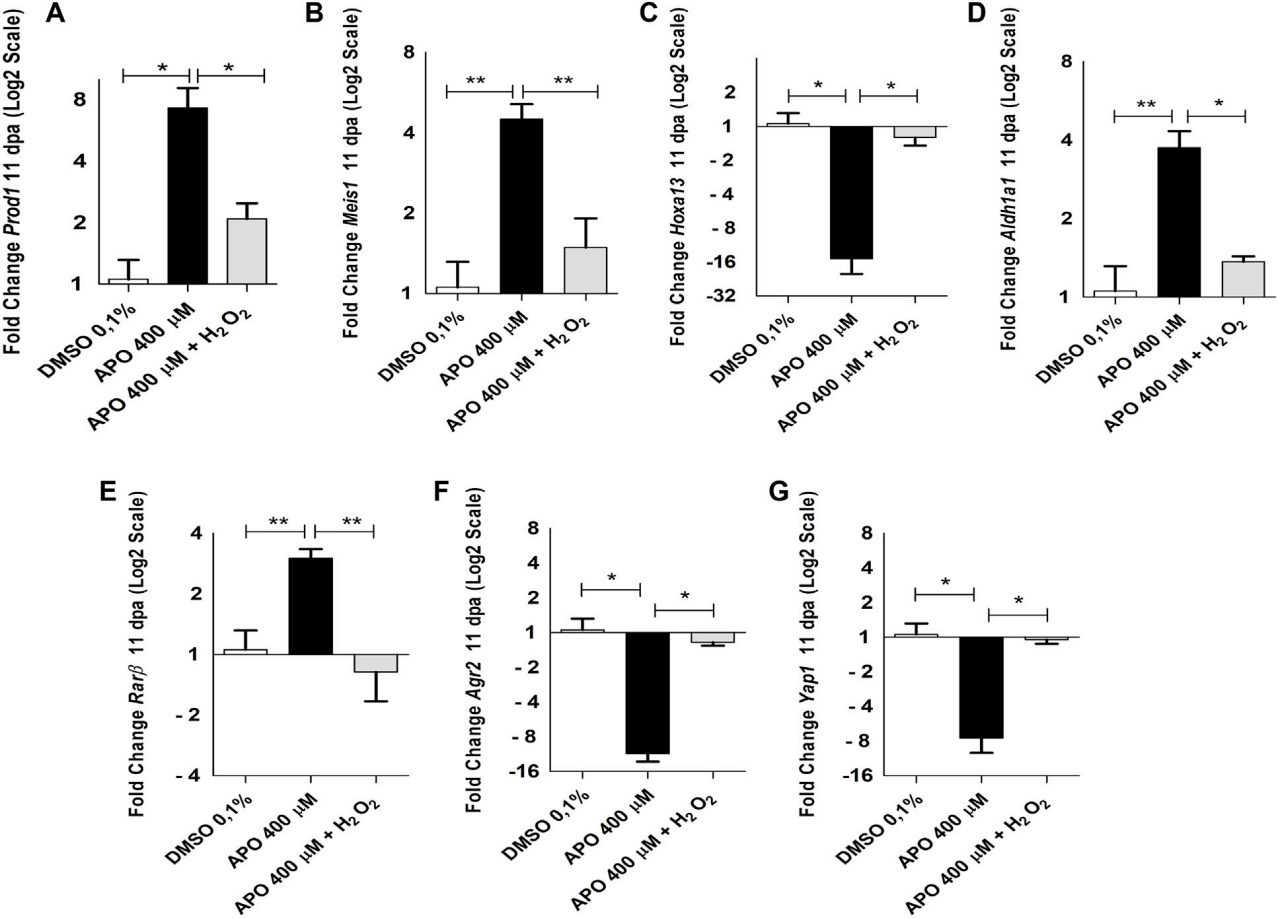
NOXs-dependent production ROS inhibition and exogenous H_2_O_2_ induces expression changes of genes related to blastema formation and positional identity during limb regeneration in *A. mexicanum* at 11 dpa. **(A–G)**, RT-qPCR of *Prod1, Meis1, Hoxa13, Aldh1a1, Rarβ, Agr2* and *Yap1* genes. The results show that blocking ROS production significantly affects the expression of the genes described here and their expression levels can be rescued by the application of exogenous H_2_O_2_. Gene expression levels were normalized to the expression of the endogenous reference gene *18S*. Data are expressed as mean ± SEM. One-way ANOVA followed by Tukey’s post hoc test was performed for comparisons between groups treated with apocynin, exogenous H_2_O_2_ and controls in 0.1% DMSO. ***p < 0.001, **p < 0.01, *p < 0.05.

The results show a significant increase in *Prod1* expression levels upon blocking ROS production compared to the control group in 0.1% DMSO. Of great relevance, rescue assay with exogenous H_2_O_2_ was able to significantly reduce *Prod1* expression to similar levels as those observed for the control group in 0.1% DMSO (Figure 7A). In addition, we also identified that like *Prod1*, *Meis1*, another gene involved in proximal identity, increased its expression post-inhibition of ROS production and its expression levels decreased upon rescue treatment with exogenous H_2_O_2_(Figure 7B). No significant effects on *Meis2* expression were observed (Supplementary Figure S3). Considering the presence of skeletal alterations in the distal region such as decrease in the number of carpals, the effect of ROS on *Hoxa13* gene expression was evaluated. In contrast to *Prod1* and *Meis1*, it was found that inhibitory treatments with apocynin significantly reduced *Hoxa13* expression levels when compared to the control group. However, rescue treatment with exogenous H_2_O_2_ rescued the expression of this gene (Figure 7C).

**FIGURE 8:**
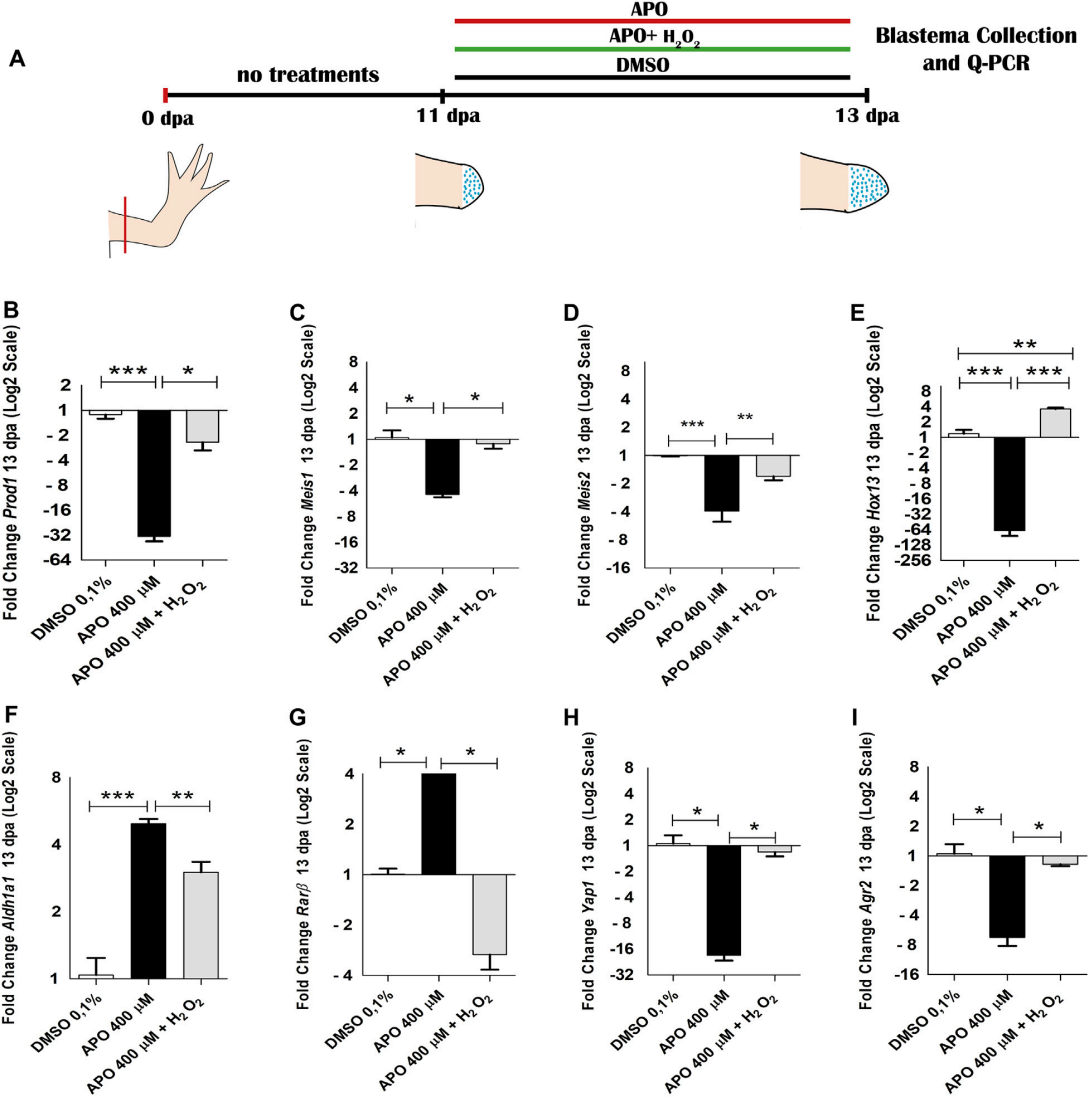
NOXs-dependent production ROS inhibition and exogenous H_2_O_2_ from 12 dpa to 13 dpa regulate the expression of positional identity and blastema formation-related genes during limb regeneration in *A. mexicanum.* **(A)**, scheme of treatments performed. Treatments used are symbolized with colored bars. **(B–I)** RT-qPCR of *Prod1, Meis1, Meis2 Hoxa13, Aldh1a1, Rarβ, Agr2* and *Yap1* genes. The results show that blocking ROS production significantly regulate the expression of these genes and their expression levels can be rescued by the application of exogenous H_2_O_2_. As in the previous trials, the expression levels were normalized to the expression of the endogenous reference gene *18S*. Data are expressed as mean ± SEM. One-way ANOVA followed by Tukey’s post hoc test was performed for comparisons between groups treated with apocynin, exogenous H_2_O_2_ and controls in 0.1% DMSO. ***p < 0.001, **p < 0.01, *p < 0.05.

On the other hand, considering the function of the retinoic acid pathway on the specification of the identity of the proximo-distal axis and previous reports linking the expression of *Prod1* and *Meis1* with this signaling pathway (Da Silva et al., 2002; Mercader et al., 2005; Kumar et al., 2015) as well as previous studies showing a regulatory effect of ROS on the activity and expression of some retinoic acid receptors (Casadevall and Sarkar, 1998; Demary et al., 2001; Park et al., 2009), we decided to evaluate the effect of ROS on the expression of some RA signaling component genes. Inhibition of ROS production generated an increase in the expression levels of *Aldh1a1* and *Rarβ* genes, which were rescued by rescue treatment with exogenous H_2_O_2_ when compared with the control group in 0.1% DMSO (Figures 7D,E). No significant differences were found in the expression of *Aldh1a2, Rarα*, and *RarG* (Supplementary Figure S3). Finally, considering previous results describing the effect of *Agr2* and *Yap1* on blastema formation and the regulation of events such as proliferation during appendage regeneration in vertebrates and their relationship with ROS production, we proceeded to evaluate their expression under conditions of loss and gain of ROS activity (Kumar et al., 2007; Hayashi et al., 2014b; Carbonell M et al., 2021). The results show that ROS inhibition significantly decreased the expression levels of *Agr2* and *Yap1* at 11 dpa. Of great interest, exogenous H_2_O_2_ treatments rescued the expression levels of these genes (Figures 7F,G). These results suggest that ROS are necessary to regulate the expression of proximo-distal identity genes. Additionally, these results also suggest *Agr2* and *Yap1* as potential mediators of redox signaling during limb regeneration in *A. mexicanum*. However, the cell population in the blastema of apocynin-treated animals was more reduced than that of the control and rescue groups which could promote a greater accumulation of proximal identity cells and consequently a higher expression of proximal identity genes as shown above (Figures 7A–E). Therefore, to corroborate whether ROS have a real effect on the expression of proximo-distal identity genes during blastema development, we allowed the blastema to grow until 11 dpa and then apply treatments with the inhibitor apocynin, as well as rescue assays with exogenous hydrogen peroxide from 12 dpa to 13 dpa (48 h of treatment), and again evaluated the impact of ROS on the expression of the genes previously evaluated (Figure 8A).

The results of this new assay show that blocking ROS production from 12 dpa to 13 dpa induced a decrease in the expression of *Prod1* and *Meis1* differing from the results obtained at 11 dpa, where apocynin treatment promoted an increase in the expression of these genes (Figures 8B,C). In addition, an increase in *Meis2* expression was now identified (Figure 8D). Like what was observed at 11 dpa, a decrease in the expression of the distal identity marker *Hoxa13* was identified (Figure 8E). On the other hand, an increase in the expression of RA pathway component genes such as *Aldh1a1* and *Rarβ* was similar to that observed at 11 dpa (Figures 8F,G). Of great relevance, H_2_O_2_ rescued the expression of each of the genes regulated by blocking NOXs activity.

Therefore, considering that the effect on the expression of proximal identity genes (*Prod1* and *Meis1*) was contrasting at 11 and 13 dpa, the data obtained suggest that the increase in the expression of these genes and the decrease in *Hoxa13* expression at 11 dpa was potentially attributed in this case, more to a predominant accumulation of proximal identity cells and a probable lack of distal identity cells as a consequence of the failure of blastema cell formation generated by the effect of ROS inhibition, than to an effect of ROS/H_2_O_2_-mediated transcriptional regulation. However, given that assays blocking ROS production from 12 to 13 dpa, when presumably proximal and distal identity cell populations are already present generated changes in the expression of several genes (including *Prod1, Meis1, Meis2, Hoxa13, Rarβ* and *Aldh1a1*) when compared to the control group, and their expression was rescued by exogenous H_2_O_2_ treatments, the data further suggest, that ROS/H_2_O_2_ does indeed regulate the expression of these proximal-distal identity genes during blastema formation and growth. Thus, ROS production is necessary for the formation of blastema cells and the expression of their proximo-distal identity genes necessary for the growth of the blastema and its derivatives. In addition to proximodistal identity genes, blocking ROS production from 12 to 13 dpa reduced the expression levels of *Yap1* and *Agr2*, which were rescued by exogenous H_2_O_2_ treatments corroborating the transcriptional regulation of these genes by ROS (Figures 8H,I).

### Inhibitory treatments of NOXs by apocynin and exogenous H_2_O_2_ treatments impact inflammatory cell recruitment and phagocytic activity

**FIGURE 9:**
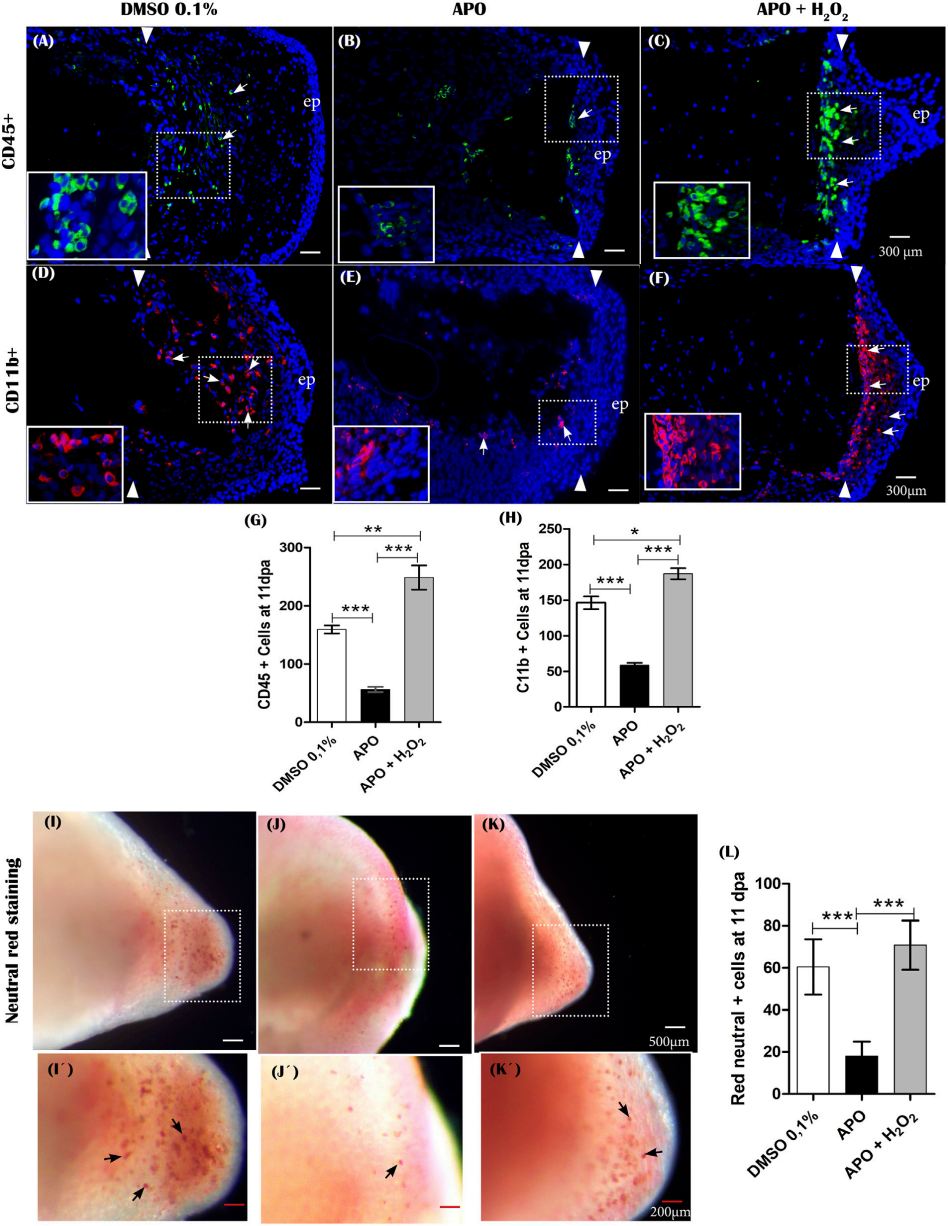
The production of ROS generated post-amputation of the limb is necessary for the recruitment and phagocytic activity of inflammatory cells. **(A–C)**, Representative immunofluorescence images against the leukocyte pan marker CD45 at 11 dpa. Boxes in white dotted lines are shown at higher magnification in solid line boxes for each image. **(A)** CD45 ^+^ cells are predominantly located in the blastema region adjacent to the amputation plane. **(B)** A reduced number of CD45 ^+^ cells are observed near the amputation plane. **(C)** a notable increase of CD45 ^+^ cells are observed in the region of the blastema and the amputation plane. **(D–F)**, Representative immunofluorescence images against CD11b. A higher presence of CD11b + cells can be seen in the control group compared with the apocynin-treated group. Animals exposed to rescue treatment show a prominent accumulation of CD11b + cells in the blastema and amputation plane. **(G,H)**, quantification of CD45 and CD11b + positive cells, respectively. **(I–K)**, vital staining with neutral red at 11 dpa. Representative images of control animals in 0.1% DMSO, exposed to apocynin inhibitor (APO) and rescue assays. Boxes in dotted white lines are shown at higher magnification in **(I′,j′,K′)** for each experimental group. **(L)**, quantification of cells positive for neutral red staining. Data are expressed as mean ± SEM. One-way ANOVA followed by Tukey’s post hoc test was performed for comparisons between groups treated with apocynin, exogenous H_2_O_2_ and controls in 0.1% DMSO. ***p < 0.001, **p < 0.01, *p < 0.05.

Inflammatory cell recruitment represents a prominent feature following tissue injury or appendage amputation in different vertebrate animal models such as zebrafish and *Xenopus* (Niethammer et al., 2009; Love et al., 2013). In zebrafish, NOX-dependent ROS production is required to promote leukocyte recruitment following tail amputation (Niethammer et al., 2009; Yoo et al., 2011). Of great interest, post-amputation of limbs in *A mexicanum*, macrophage recruitment is required for proper regeneration of this structure (Godwin et al., 2013), and previous studies during tail regeneration in this animal model have shown that NADPH oxidase-dependent ROS production is required to promote leukocyte recruitment (Carbonell M et al., 2021). In addition, several studies have shown that apocynin possesses anti-inflammatory properties mediated by the regulation of NADPH oxidases and oxidative stress, which has been explored in several inflammatory diseases. (Kim et al., 2012; Hwang et al., 2019; Boshtam et al., 2021). This background motivated us to question whether inhibitory treatments of ROS production using the NOX inhibitor apocynin have any effect on the inflammatory response after limb amputation. To address this question, we first evaluated the effect of blocking ROS production from 0 dpa to 11 dpa on leukocyte recruitment using a leukocyte pan marker, CD45 (Im et al., 2011). The results show a significant reduction in CD45 ^+^ leukocyte recruitment in animals treated with the inhibitor apocynin compared to controls in DMSO (Figures 9A,B,G). Of great interest, rescue treatment with exogenous H_2_O_2_ rescued the recruitment of these cells and even promoted higher recruitment of CD45 ^+^ cells when compared to controls in 0.1% DMSO (Figures 9C,G). The CD45 ^+^ cells were predominantly located in the region underlying the amputation plane (Figures 9A–C). Subsequently, to evaluate the effect of blocking ROS production on monocyte/macrophage recruitment, we used the CD11b marker, previously used in this animal model for the same purpose (Godwin et al., 2013). Similar to what was observed previously, treatment with apocynin reduced the number of CD11b + cells compared to the control group, and relevantly, treatment with exogenous H_2_O_2_ promoted a greater increase in monocyte/macrophage recruitment compared to both the group exposed to the inhibitory treatment and the control group in DMSO (Figures 9D–F,H). Finally, we set out to evaluate the effect of inhibitory treatment with apocynin on macrophage phagocytic activity using the neutral red labeling method previously used in this animal model (Godwin et al., 2013; Franklin et al., 2017). The results show a remarkable accumulation of phagocytic cells in the blastema of control animals in 0.1% DMSO (Figures 9I, 9I’). In contrast, treatment with apocynin reduced the number of phagocytes (Figures 9J,J’,L). On the other hand, treatment with exogenous H_2_O_2_ rescued the number of phagocytic cells, which were widely located in the blastema region (Figures 9K,K’,L). Consistently, neutral red-positive cells were located at sites comparable to CD11b + cells. In the first instance, these results suggest that NADPH oxidase-dependent ROS production is required for the recruitment and activity of inflammatory cells whether leukocytes or monocytes/macrophages. This then suggests that the inflammatory response represents a potential mechanism for mediating ROS function during limb regeneration in *A. mexicanum*.

## Discussion

### ROS production and signaling are conserved during the regeneration of appendages such as tail and limbs in vertebrates

Recently, ROS have emerged as a group of key molecules in redox signaling, among them H_2_O_2_ (Sies, 2017; Sies and Jones, 2020b). ROS are highlighted because they can regulate a wide range of cellular processes including proliferation, migration, differentiation, and apoptosis, among others (Milkovic et al., 2019; Sies and Jones, 2020b; Sun et al., 2020). This has promoted a growing interest in the requirement of these molecules during biological processes such as the regeneration of tissues and complex structures such as appendages, including tails and limbs in vertebrates (Meda et al., 2017, 2018; Rampon et al., 2018). Our results show that post-amputation of limbs in axolotl, ROS production was detected up to 18 dpa, with peaks of production at 2, 7, and 11 dpa which correlates with events such as wound closure, blastema formation, and growth. These results are comparable with previous results obtained in our group where after tail amputation in juvenile salamanders, ROS production was also detected at these stages of the regenerative process (Carbonell M et al., 2021). Similarly, another study performed in embryos of the same species shows that ROS production is also detected during wound closure and blastema formation (Al Haj Baddar et al., 2019). In the present study, NOX-dependent blockade of ROS production affected the size and patterning of the regenerated limb. Consistent with these results, blocking ROS production during tail regeneration in embryonic and juvenile axolotls also perturbed regeneration of this structure (Al Haj Baddar et al., 2019). This suggests that NOX-dependent ROS signaling represents a conserved mechanism during regeneration of appendages such as tail and limb in the salamander *A. mexicanum*. On the other hand, studies in other vertebrate species show that post-amputation of tail in *Xenopus*, Gecko, and tail fin in zebrafish, ROS production is also part of an immediate response which is sustained during the early stages of the regeneration process (Gauron et al., 2013; Love et al., 2013; Ferreira et al., 2016, 2018; Meda et al., 2016; Zhang et al., 2016; Romero et al., 2018). Similarly, blocking ROS production during regeneration in these species disrupts the regeneration of the amputated structure. Additionally, studies in *Xenopus* tadpoles during stages 52–53 show that ROS production is necessary for regeneration of amputated limbs (Zhang et al., 2018). Taken together, our results suggest that NOXs-dependent ROS/H_2_O_2_ signaling is a conserved mechanism during appendage regeneration among different vertebrate species with regenerative capacity, and in particular, its production is required during limb regeneration in urodele amphibians such as axolotls, and other amphibians such as *Xenopus*.

### NOX-dependent ROS production is necessary for cell cycle re-entry and blastema formation during limb regeneration

Blastema formation and growth are critical steps during epimorphic limb regeneration (Tassava et al., 1987; McCusker et al., 2015; Stocum, 2019). Thus, the recruitment of progenitor cells from remnant “Stump” tissues and the re-entry of these cells into the cell cycle are necessary events for blastema development (Tassava et al., 1987; Heber-Katz et al., 2013; McCusker et al., 2015). In the present study, blocking NOX-dependent ROS production from 0 dpa to 11 dpa affected blastema formation (failure of blastema progenitor recruitment) and significantly decreased BrdU incorporation, reflecting a reduction in cell cycle reentry. Consistent with our results, previous studies show that blocking ROS production during tail regeneration in embryonic and juvenile axolotls disrupts blastema formation and reduces cell proliferation (Al Haj Baddar et al., 2019). Additionally, studies in other vertebrates such as *Xenopus* and zebrafish show that ROS production induced post-amputation of tail and tail fin is necessary to promote blastema cell formation and proliferation (Yoo et al., 2012; Gauron et al., 2013; Love et al., 2013). Relevantly, our results show that the application of exogenous H_2_O_2_ rescues blastema formation and increases cell proliferation levels in limbs exposed to the inhibitor apocynin. These findings are consistent with previous results showing that overexpression of *cyba*, an inducer of ROS production, rescues blastema formation inhibited by mcr4 receptor activity-dependent reduction in ROS production during limb regeneration in *Xenopus* (Zhang et al., 2018). Similarly, during tail regeneration in this animal model, H_2_O_2_ production induced by *cyba* overexpression rescues blastema formation (Love et al., 2013). Like our results, the application of exogenous H_2_O_2_ rescues blastema formation, proliferation, and growth inhibited by NOXs complex blockade during tail regeneration in axolotls (Carbonell M et al., 2021). On the other hand, during zebrafish tail fin regeneration, exogenous H_2_O_2_ treatments rescue cell proliferation levels and blastema formation in denervated fins or fins exposed to SHH signaling inhibitors (Meda et al., 2016; Thauvin et al., 2022). Accordingly, our results suggest that ROS, particularly H_2_O_2_, are required as early signals for blastema formation and re-entry into the cell cycle of blastema progenitors during limb regeneration in axolotl like that observed in other vertebrates.

To date, the molecular and cellular mechanisms by which ROS/H_2_O_2_ regulate blastema formation and proliferation in vertebrates are still under study and a small number of studies show some evidence of interactions between ROS and other signaling pathways (Yoo et al., 2012; Gauron et al., 2013; Love et al., 2013; Romero et al., 2018; Thauvin et al., 2022). Our results show that inhibition of ROS production generated a reduction in *Agr2 (nAG)* and *Yap1* expression at 11 dpa during early/mid blastema formation, positioning these genes as new potential mediators of ROS signaling. Of great interest, the expression of these genes was rescued post-treatment with exogenous H_2_O_2_. These results agree with previous studies, where post-amputation of tails in axolotls, similar treatments generate a similar effect on *Agr2* and *Yap1* expression during early blastema formation (Carbonell M et al., 2021). Additionally, in this animal model, blocking ROS-dependent YAP1 signaling decreases cell cycle re-entry and mitotic index, affecting blastema formation and growth (Carbonell M et al., 2021). Of great relevance, during limb regeneration in axolotls, YAP1 has been detected at the nuclear level in proliferating blastema dedifferentiated cells, and blocking its activity, delays blastema formation and growth, like what was observed in the present study by blocking ROS production (Erler, 2017). Likewise, YAP1 activity is necessary to promote blastema proliferation and formation during tail and limb regeneration in *Xenopus* (Hayashi et al., 2014b; 2014a). In addition, several studies demonstrate that Yap1 expression and transcriptional activity is regulated by ROS, particularly H_2_O_2_ (Delaunay et al., 2000; Veal et al., 2003; Dixit et al., 2014; Tung et al., 2018; Shome et al., 2020). Regarding *Agr2*, consistent with our results, previous studies show that ROS can regulate its expression in regeneration and cancer contexts (Zweitzig et al., 2007; Chevet et al., 2013; Carbonell M et al., 2021) and nAG can promote regeneration of denervated limbs, as well as induce a high mitogenic response on blastema cells in newts (Kumar et al., 2007; Grassme et al., 2016). Therefore, our results and the above results suggest that ROS signaling could potentially be mediated by YAP1 and AGR2 to induce blastema formation during limb regeneration.

Finally, taking into account the localization of ROS production detected in this study and that its blockade led to alterations in gene expression and failure of blastema formation, and the fact that in salamanders signals from the epithelium and from the blastema itself are necessary to induce its formation and growth, respectively (Mullen et al., 1996; Han et al., 2001; Christensen et al., 2002; Stocum, 2017; Lovely et al., 2022), we propose two potential ways by which ROS can regulate the expression and potential activity of signals required for blastema formation. 1) according to the remarkable ability of ROS, especially hydrogen peroxide to diffuse with great ease across membranes (Veal et al., 2007; Miller et al., 2010; Bienert and Chaumont, 2014; Sies, 2017; Ledo et al., 2022), it could potentially regulate the expression and activity of genes and signaling pathways directly on blastema cells at the expense of its outstanding diffusion capacity from the epithelium to the blastema and between cells of the same blastema. 2) On the other hand, although in our work we did not evaluate the effect of ROS on the activity and expression of genes and activation of signaling pathways with epithelial predominance, ROS could also regulate the expression of soluble factors from the epithelium (AEC), which in turn can influence signaling pathways in the remaining tissue and the blastema itself to regulate its formation and growth. Additionally, considering that the localization of ROS was persistent in the AEC and the blockade of NOXs only slowed the rate of blastema formation without completely blocking its formation, then the function of the AEC as an inducer of blastema formation was not blocked in its entirety but transiently. Further studies are required to evaluate the effect of ROS on the expression and activity of epithelial and mesenchymal localized genes in the blastema.

### ROS/H_2_O_2_ an early signal potentially involved in the determination of regenerated limb size

The regulation of growth and determination of the final size of a developing structure or during regeneration represents an area of great interest and the underlying mechanisms are still debated (Hafen and Stocker, 2003; Tornini and Poss, 2014; Penzo-Mendez and Stanger, 2015; Vollmer et al., 2017; Wells et al., 2022). Our results show that blocking ROS production during the early stages of regeneration (early/mid blastema formation) generated a significant shortening in the final size of the regenerate and its skeletal components, simulating a miniature limb. We hypothesize that a first potential explanation for this phenotype can be attributed to the formation of a reduced blastema size caused by decreased cell cycle re-entry and decreased amount of progenitor cells post-blockade of ROS production. This approach is consistent with previous results showing that the reduction in proliferation levels and progenitor cell numbers in axolotl limb buds by colchicine results in the development of miniature limbs as a product of a decrease in the embryonic field “limb bud area" (Alberch and Gale, 1983). Therefore, the reduction in this embryonic field could be equivalent to the decrease in blastema size observed in the present study. Similarly, other studies have shown that repeated amputations during limb regeneration in axolotls generate a miniature limb phenotype, which developed from a reduced blastema size, similar to that observed in our study (Bryant et al., 2017). Of great interest, the authors showed that re-amputation of the miniaturized limb generates a reduced size blastema and consequently, a new miniaturized limb, suggesting that local blastema tissue size and cellular bias represent a major force for size determination during regeneration (Bryant et al., 2017). Supporting our approach, similar to our results, blocking ROS production during regeneration of other appendages such as tail in salamanders, *Xenopus*, Gecko, and tail fin in Zebrafish reduces the size of the regenerated appendage (Gauron et al., 2013; Love et al., 2013; Zhang et al., 2016; Carbonell M et al., 2021). Accordingly, our results suggest that ROS production is necessary as an early signal to promote an adequate number of progenitor cells in the blastema to support its growth and the final size of the regenerated structure.

Several studies show that from early stages the presence of the nerve is necessary to promote blastema formation and growth, and denervation of a mid-stage blastema (Singer, 1978; Mullen et al., 1996; Stocum, 2019), as well as blockade of early nerve-regulated signals (Farkas et al., 2016; Satoh et al., 2016; Purushothaman et al., 2019) result in miniature limbs, indicating that from early stages the nerve and other signals regulate the size of the regenerate. Wells *et al* demonstrated that nerve fiber thickness as well as factors produced from the nerve are determinants from the early tiny stage in regulating the growth rate and determining the final size of the regenerated limb (Wells et al., 2021). Similar to the requirement of the nerve to regulate the size of the regenerate, our results show that ROS blockade from 0 dpa to 11 dpa, affects the final size of the regenerated limb. Additionally, this phenotype is rescued by the addition of exogenous H_2_O_2_ and relevantly, previous reports have suggested an integration between ROS signaling and nerve (Meda et al., 2018). Therefore, it is feasible to hypothesize that ROS production could regulate the production of factors from the nerve affecting the size of the regenerate. Previous studies in Zebrafish show that ROS production regulates *Shha* expression in Schwann cells and reciprocally, HH (shha) signaling from these cells controls ROS levels and rescues the size of the regenerated caudal fin in animals treated with ROS production inhibitors (Meda et al., 2016; Thauvin et al., 2022). Of great relevance, our results show that blocking ROS production and exogenous H_2_O_2_ affect *Agr2* expression levels, which has been previously detected in Schwann cells during limb regeneration in axolotls (Kumar et al., 2010) and overexpression of this gene promotes blastema formation and denervated limb regeneration in newts (Kumar et al., 2007). Notably, limb denervation in *Xenopus* reduces the ROS production required for the regeneration of this structure and *Agr2* inhibition reduces the regenerated tail area in this same animal model (Zhang et al., 2018; Ivanova et al., 2021). Additionally, during tail regeneration in salamanders, blocking ROS production reduces *Agr2* expression levels and the final size of the regenerate (Carbonell M et al., 2021). Therefore, these findings suggest that from early stages ROS could regulate the production of *Agr2* from the Schwann cells, without excluding the production of other factors such as Shh, Fgf, Wnt and Bmp also necessary for limb regeneration (Mullen et al., 1996; Kawakami et al., 2006; Guimond et al., 2010; Wischin et al., 2017; Purushothaman et al., 2019) and regulated by ROS during the regeneration of other appendages (Love et al., 2013; Meda et al., 2016; Satoh et al., 2016; Romero et al., 2018) to favor an adequate regenerative response that contributes to growth control and size of the regenerate.

On the other hand, several of the genes regulated by blocking ROS production, such as *Prod1* and *Yap1*, have been previously implicated in the regeneration of shortened structures. The shortening of skeletal structures of regenerated limbs in axolotl has also been evidenced after overexpression of *Prod1*, which also reduces proliferation levels in the blastema (Echeverri and Tanaka, 2005). Consistent with our results, *Yap1* expression and its transcriptional activity are regulated by ROS/H_2_O_2_ production (Delaunay et al., 2000; Veal et al., 2003; Kim and Hahn, 2013; Tung et al., 2018; Shome et al., 2020), and of great interest, its activity has been implicated in the control of organ and appendage size in contexts such as development and generation (Camargo et al., 2007; Dong et al., 2007; Halder and Johnson, 2011; Hayashi et al., 2015). Particularly, during tail regeneration in *Xenopus* and axolotls, blocking *Yap1* transcriptional activity causes regeneration of reduced size tails and in the axolotls model, its activity was dependent on ROS production (Hayashi et al., 2014a). Considering the above, our results suggest that ROS signaling can regulate growth and size determination through Yap1 signaling and Prod1 during limb regeneration. Furthermore, although in our study we did not evaluate the effect of ROS on tissue differentiation, a premature differentiation of tissues with a reduction in tissue growth is not excluded as a potential mechanism to explore as a cause for this phenotype. Accordingly, our results propose ROS signaling as a new candidate for regulating regenerate size early in the regenerative process, potentially participating in blastema patterning and proliferation. Further studies are required to elucidate the mechanisms proposed here.

### Regulation of positional identity genes by ROS and their potential relationship to skeletal defects generated post-inhibition of NADPH oxidases

Our results show that blockade of ROS production from 0 dpa to 11 dpa generated a series of skeletal alterations that included in addition to proximal-distal shortening of skeletal structures: failure of integration between the regenerated structure and the remnant tissue, and decrease in the number of carpals, and alteration in joint morphogenesis. Likewise, blocking ROS production generated overexpression of gene *Meis1,Meis2, Prod1, Rarβ*, and *Aldh1a1*, and a reduction in the expression of the *Hoxa13*. Of great relevance, both skeletal alterations and expression levels of these genes were rescued by exogenous H_2_O_2_ treatments, suggesting that ROS/H_2_O_2_ production from early stages is required for the final patterning of skeletal structures and its function, may potentially be mediated by the activity of these positional identity genes.

Comparable to our results, integration failures have been reported when regenerating limbs are exposed to different exogenous treatments such as vitamin D and RA (Niazi et al., 1985; McCusker et al., 2014; Vieira et al., 2018). The application of exogenous RA during early/late blastema formation generates ectopic integration characterized by hypertrophic growth and a discontinuity in the area of integration between the newly regenerated humerus and the remnant tissue, similar to what was observed in our study when ROS production is blocked (Niazi et al., 1985). Although to date, the mechanisms responsible for the failure of integration have not been elucidated, several authors suggest that a potential explanation may be related to disturbances in positional identity (McCusker and Gardiner, 2014; Vieira and McCusker, 2018). Accordingly, overexpression of several proximal identity genes such as *Mesi1, Prod1, Rarβ*, and *Aldh1a1* after ROS inhibition may, on the one hand, affect the positional values of proximal cells that potentially contribute to the integration between the new regenerate and the remaining tissues and, on the other hand, may promote some degree of proximalization of more distal cells, which by preserving part of their distal identity, may affect integration by a “discontinuity of positional values” as has been previously proposed (McCusker and Gardiner, 2014).

Accordingly, ROS function could be mediated by RA and previous studies have shown a relationship between the redox state and RA signaling (Casadevall and Sarkar, 1998; Demary et al., 2001; Cao et al., 2015). Studies performed in melanoma tumor cell lines and fibroblasts show that antioxidant treatments or overexpression of antioxidant enzymes increase the binding of RA receptors (RARa/RARβ) to RARE (Retinoic Acid Response Elements) and the application of exogenous H_2_O_2_ stabilizes RA signaling by decreasing the affinity of its receptors to RARE (Demary et al., 2001; Park et al., 2009). Additionally, *Rarβ* expression levels are increased by reducing ROS levels and rescued post-application of exogenous H_2_O_2_, similar to what was observed in our results (Park et al., 2009). Of great interest, during AR-dependent nephrogenesis in *Xenopus,* peroxiredoxin 1(Prdx1) functions as a modulator of AR signaling by regulating ROS levels (Chae et al., 2017). Therefore, we hypothesize that optimal levels of ROS are necessary to maintain balanced RA signaling (including RARa) and consequently, the expression of other genes such as *Meis1, Meis2* and *Prod1* for the establishment of positional identity values that favor integration between the regenerated structure and the remnant tissue during limb regeneration in axolotls.

On the other hand, inhibition of ROS production also generated decrease in the number of carpals and alterations in elbow joint morphogenesis. Previous studies show that the specification of intersegmental precursor cells (stylopod, zeugopod, and autopod) during limb regeneration in axolotls is progressively defined during blastema formation and growth (from early, middle, to late blastema) and follows a pattern similar to that reported during development (Echeverri and Tanaka, 2005; Roensch et al., 2013). Previous studies show that this specification is regulated by HoxA family genes. Particularly Hoxa13-positive cells located more distally in the mid blastema are determined to form autopod structures (Gardiner and Bryant, 2004; Roensch et al., 2013). Our results show that ROS blockade during early/mid blastema formation reduced *Hoxa13* gene expression and generated a decrease in the number of carpals; additionally, these alterations and *Hoxa13* expression were rescued by the application of exogenous H_2_O_2_. Although there are no previous reports evidencing transcriptional regulation of Hox genes by ROS, a previous study shows that the transcriptional activity of the HoxB5 factor is dependent on its ROS-mediated oxidation (Galang and Hauser, 1993). Therefore, this background and our results suggest that ROS, specifically H_2_O_2_, could regulate distal cell identity by mediating activity and *Hoxa13* factor expression for the correct patterning of blastema cells destined to form distal structures such as carpal bones either directly or indirectly through RA signaling. Additionally, the phenotype of reduced carpal number could be due to decreased levels of cell proliferation, or potentially, to a differentiation defect, such as “premature differentiation” as proposed above for skeletal shortening, which would not allow the formation of the correct number of carpals. Further trials evaluating this approach are needed.

### ROS production as a potential regulatory mechanism of the inflammatory response during limb regeneration

Following amputation of appendages in different vertebrate models, several responses of adjacent tissues have been identified (Yoo et al., 2012; Ferreira et al., 2016; Liu et al., 2021). Within these responses, ROS production and activation of the inflammatory response top the list of major events (Niethammer et al., 2009; Yoo et al., 2011; Godwin et al., 2013; Meda et al., 2018; Niethammer, 2018; Al Haj Baddar et al., 2019; Carbonell M et al., 2021; Liu et al., 2021).

Our results show that ROS production is required for blastema formation and proper regeneration of the limb. Additionally, our data show that blocking ROS production affects the recruitment of cells such as leukocytes (CD45^+^) including CD11b + monocytes/macrophages, as well as phagocytic activity. The relationship between ROS production and the inflammatory response has been described in principle under the fact that polymorphonuclear leukocytes generate ROS from NADPH oxidase activity as an antimicrobial mechanism (Gabig and Babior, 1979; Panday et al., 2015). However, in the context of regeneration, the functional relationship between ROS production and the inflammatory response is still unclear. Previous studies in Zebrafish show that NADPH oxidase Duox-dependent ROS production is necessary to promote leukocyte recruitment favoring their directionality and tissue infiltration during tail regeneration (Niethammer et al., 2009). On the other hand, in adult zebrafish and larvae it has been identified that H_2_O_2_ production from the wound epithelium is necessary to activate the redox sensor protein Lyn in neutrophils favoring the recruitment of these cells (Yoo et al., 2011). Previous results in our group have shown that during tail regeneration in *A mexicanum* blocking ROS production reduces leukocyte recruitment to the site of blastema formation (Carbonell M et al., 2021). Therefore, our results and the aforementioned studies suggest that ROS production acts as a necessary signal to promote post-amputation inflammatory cell recruitment of appendages such as tails and limbs. This function would be predominantly regulated from the wound epithelium and AEC from where the main source of ROS concerning limb regeneration in *A mexicanum* was identified. However, although the inflammatory response has been suggested as a determinant factor for the regenerative response, several studies show some contrasting results among vertebrates (Godwin and Brockes, 2006; Mescher and Neff, 2006; Godwin et al., 2017). Studies in Zebrafish show that immune system cell depletion does not affect caudal and tail fin regeneration in embryos and adults of this species (Mathew et al., 2007; Yoo et al., 2012). On the other hand, similar results have been reported in the *Xenopus* model, where myeloid precursor depletion does not affect tail regeneration (Love et al., 2013). Thus, although in each of these models ROS production is necessary to promote regeneration and recruitment of these cells, in these species it appears that the inflammatory response is not the mechanism by which ROS mediate their role in the regenerative response. However, in other vertebrate models, the recruitment of inflammatory cells is required for the regenerative response. Studies in *A mexicanum* show that monocyte/macrophage recruitment is required for limb regeneration (Godwin et al., 2013). Thus, early macrophage depletion blocks regeneration and promotes scar tissue formation between the remnant tissue and the wound epithelium affecting blastema formation. On the other hand, macrophage depletion at a later stage generates a delay in regeneration and reduce the expression of *Mmp9/Mmp3* and failure of extracellular matrix remodeling. Additionally, it reduces the expression of blastema markers such as *Prrx1* and *Sp9,* as well as reduces the activation of TGFβ, necessary for blastema formation and progression of the regenerative response (Godwin et al., 2013). Interestingly, our results show that blocking ROS production, in addition to generating a decrease in monocyte/macrophage (CD11b+) recruitment and a reduction in their phagocytic activity, affected blastema formation. ROS blockade promoted the formation of a fibrous tissue between the epithelium and the remnant tissue, similar to that reported when macrophage depletion is induced (Godwin et al., 2013). These findings suggest that in the case of limb regeneration in *A mexicanum*, regulation of the inflammatory response could represent a potential mechanism by which ROS may be mediating processes such as extracellular matrix remodeling necessary for blastema formation. In support of this, in contexts other than regeneration such as cancer, ROS have been implicated during extracellular matrix remodeling by mediating the expression of several metalloproteinases (Svineng et al., 2008; Shin et al., 2015; Hsieh et al., 2017). Further assays are needed to assess whether ROS blockade affects the activity of the metalloproteinases MMP9 and MMP3, as well as the expression and activation of other pathways regulated by macrophage activity during limb regeneration in the *A mexicanum* model.

## Conclusion

**FIGURE 10:**
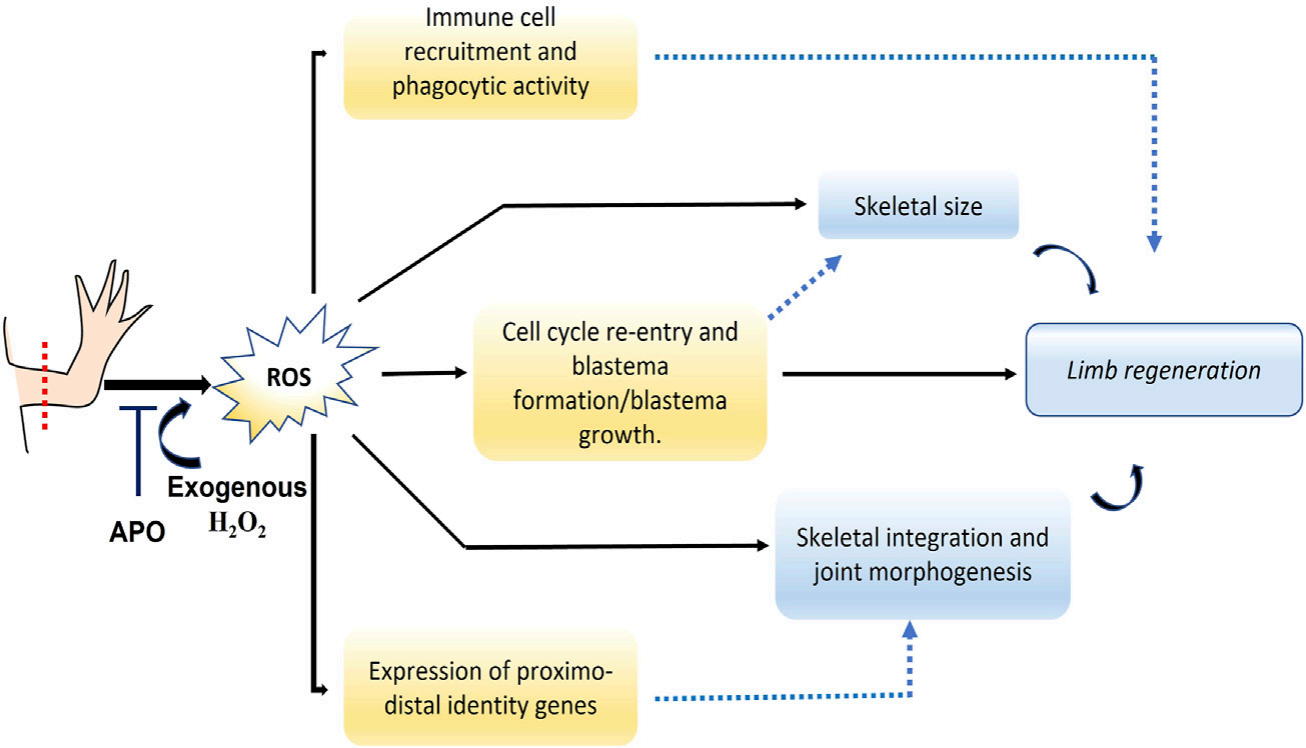
Graphical abstract of the results obtained and putative mechanistic insight of ROS in regeneration. Following limb amputation, ROS are produced. The production of ROS is necessary for the re-entry into the cell cycle of the remaining tissues and consequently for the formation and growth of the blastema. ROS also regulates the expression of genes previously involved in blastema formation such as *Yap1* and *Agr2*. This suggests that ROS production-dependent blastema size influences the final size of the limb and its regenerating skeleton. Moreover, ROS regulates the expression of proximal-distal identity genes, suggesting that the obtained phenotype of integration failure between the regenerated and remnant skeleton as well as alterations in joint morphogenesis could be regulated by ROS production. In addition, ROS are necessary to promote the recruitment of inflammatory cells such as leukocytes including monocytes/macrophages during blastema formation, as well as their phagocytic activity. Therefore, we hypothesize that the regulation of the inflammatory response represents a potential mechanism by which ROS mediate its function during axolotl limb regenearation. The solid black lines represent the results obtained in this work and the dotted blue lines represent the proposed relationships according to previous studies and those obtained in this work.

Overall, our results propose ROS, particularly H_2_O_2_ as an early signal necessary for proper limb regeneration in salamanders, mediating in the first instance the formation and growth of the blastema. Likewise, it was evidenced that ROS/H_2_O_2_ are necessary for the correct morphogenesis and size of the skeletal structures, as well as for the correct integration between the new regenerated structure and the remaining tissue. Finally, according to the transcriptional regulation evidenced in the present work, the function of ROS/H_2_O_2_ would be potentially related to the proximal-distal specification of intersegmental precursors for stylopod, zeugopod, and autopod formation. Additionally, our results show for the first time that ROS are necessary to promote the recruitment of inflammatory cells such as leukocytes including monocytes/macrophages during blastema formation, as well as their phagocytic activity (Figure 10). Future studies are needed to further decipher the mechanism by which ROS such as H_2_O_2_ participate in limb regeneration and how their activity relates to signaling pathways involved in proximal-distal and anterior-posterior specification during limb regeneration.

## Data Availability

The original contributions presented in the study are included in the article/Supplementary Material, further inquiries can be directed to the corresponding authors.
