# Supplementary material for: Post-amputation reactive oxygen species production is necessary for axolotls limb regeneration
Source: Front Cell Dev Biol. 2022 Aug 26;10:921520. doi: 10.3389/fcell.2022.921520 (PMC9458980; doi:10.3389/fcell.2022.921520)
Supplement: Supplementary file 1 [file DataSheet1.docx]

Supplementary Material


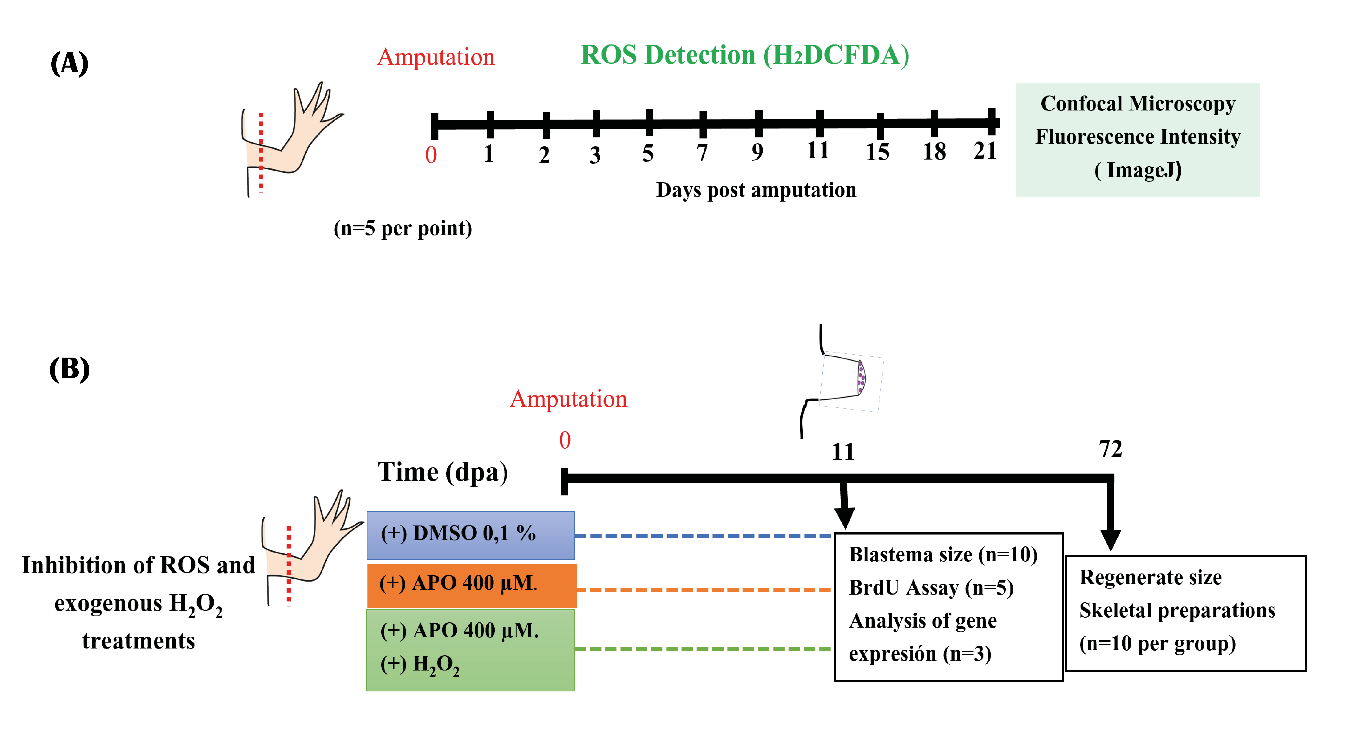


**Supplementary Figure 1**. Overall experimental design. a) Detection of ROS production . Animals were incubated in DCFDA for 2 h, subsequently washed in 20% Holftreter solution, anesthetized and photographed under confocal microscopy. Five animals were used for each day of analysis. The animals of each point were independent of the other points of analysis. B) ROS production blocking experiments and rescue assays with exogenous hydrogen peroxide. Analysis of blastema formation, cell cycle re-entry and expression of genes of interest were performed at 11 dpa. Additionally, skeletal analysis and regenerated limb size were evaluated at 72 dpa.


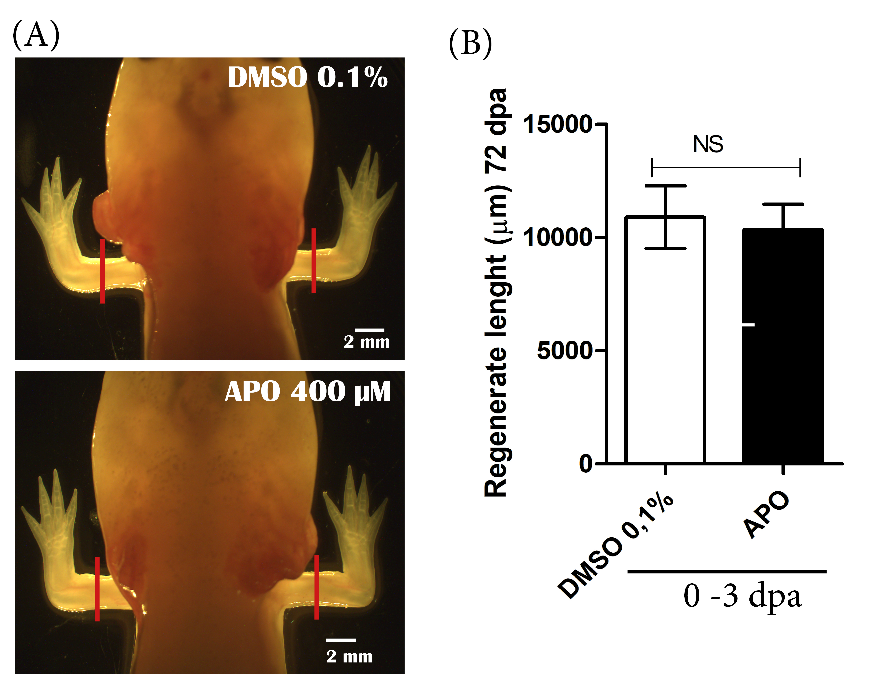


**Supplementary Figure 2.** Blockade of ROS production from 0 dpa to 3 dpa does not affect limb regeneration. **(A)**, Representative image of control and apocynin-treated animals. Apocynin treatment does not induce alterations in morphogenesis and size of the regenerated limb. **(B)**, Quantification of regenerated limb size. No statistically significant differences were observed between the control group in DMSO and apocynin-treated animals. An unpaired t-student test was performed. P<0.05 was considered statistically significant. Data are expressed as mean ± SEM. ***P < .001, **P < .01, *P < .05.


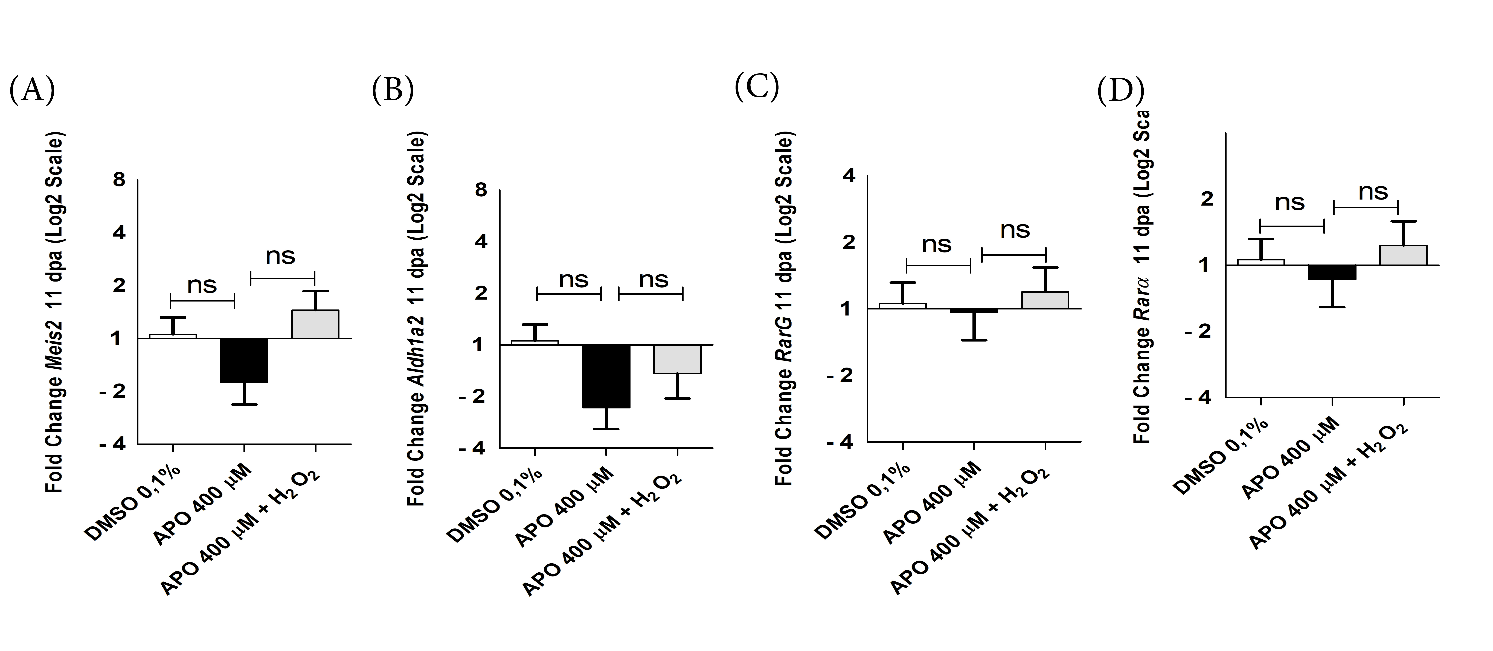


**Supplementary Figure 3.** NOXs-dependent ROS inhibition and exogenous H_2_O_2_ do not affect the expression of *Meis2, Aldh1a2, RARG, RARα* genes. (A - D), RT-qPCR of *Meis2, Aldh1a2, RARG, RARα* genes. Gene expression levels were normalized to the expression of the endogenous *18S* reference gene. Data are expressed as mean ± SEM. One-way ANOVA followed by Tukey's post hoc test was performed for comparisons between groups treated with apocynin, exogenous H_2_O_2,_ and controls in 0.1% DMSO. ***P < .001, **P < .01, *P < 0.05
